# Deep sequencing transcriptional fingerprinting of rice kernels for dissecting grain quality traits

**DOI:** 10.1186/s12864-015-2321-7

**Published:** 2015-12-21

**Authors:** Chiara Biselli, Paolo Bagnaresi, Daniela Cavalluzzo, Simona Urso, Francesca Desiderio, Gabriele Orasen, Alberto Gianinetti, Federico Righettini, Massimo Gennaro, Rosaria Perrini, Manel Ben Hassen, Gian Attilio Sacchi, Luigi Cattivelli, Giampiero Valè

**Affiliations:** CREA- Council for Agricultural Research and Economics, Rice research unit, S. S. 11 to Torino Km 2,5, Vercelli, 13100 Italy; CREA - Council for Agricultural Research and Economics, Genomics Research Centre, Via S. Protaso 302, Fiorenzuola d’Arda (PC), 29017 Italy; DiSAA - Department of Agricultural and Environmental Sciences, Università degli Studi di Milano, Via G. Celoria 2, Milan, 20133 Italy

**Keywords:** Rice, Caryopsis, Grain quality, RNA-Seq, Co-regulation, SNP

## Abstract

**Background:**

Rice represents one the most important foods all over the world. In Europe, Italy is the first rice producer and Italian production is driven by tradition and quality. All main rice grain quality traits, like cooking properties, texture, gelatinization temperature, chalkiness and yield, are related to the content and composition of starch and seed-storage proteins in the endosperm and to grain shape. In addition, a number of nutraceutical compounds and allergens are known to have a significant effect on grain quality determination. To investigate the genetic bases underlying the qualitative differences that characterize traditional Italian rice cultivars, a comparative RNA-Seq-based transcriptomic analysis of developing caryopsis was conducted at 14 days after flowering on six popular Italian varieties (Carnaroli, Arborio, Balilla, Vialone Nano, Gigante Vercelli and Volano) phenotypically differing for qualitative grain-related traits.

**Results:**

Co-regulation analyses of differentially expressed genes showing the same expression patterns in the six genotypes highlighted clusters of *loci* up or down-regulated in specific varieties, with respect to the others. Among them, we detected *loci* involved in cell wall biosynthesis, protein metabolism and redox homeostasis, classes of genes affecting in chalkiness determination. Moreover, *loci* encoding for seed-storage proteins, allergens or involved in the biosynthesis of specific nutraceutical compounds were also present and specifically regulated in the different clusters. A wider investigation of all the DEGs detected in pair-wise comparisons revealed transcriptional variation, among the six genotypes, for quality-related *loci* involved in starch biosynthesis (e.g. *GBSSI*, starch synthases and *AGPase*), genes encoding for transcription factors, additional seed storage proteins, allergens or belonging to additional nutraceutical compounds biosynthetic pathways and *loci* affecting grain size. Putative functional SNPs associated to amylose content in starch, gelatinization temperature and grain size were also identified.

**Conclusions:**

The present work represents a more extended phenotypic characterization of a set of rice accessions that present a wider genetic variability than described nowadays in literature. The results provide the first transcriptional picture for several of the grain quality differences observed among the Italian rice varieties analyzed and reveal that each variety is characterized by the over-expression of a peculiar set of *loci* affecting grain appearance and quality. A list of candidates and SNPs affecting specific grain properties has been identified offering a starting point for further works aimed to characterize genes and molecular markers for breeding programs.

**Electronic supplementary material:**

The online version of this article (doi:10.1186/s12864-015-2321-7) contains supplementary material, which is available to authorized users.

## Background

Rice is the staple food for nearly half of the world’s population. The world *per capita* rice consumption in 2013–2014 was 57.3 kg/yr of milled rice (http://www.statista.com/statistics/256002/global-per-capita-rice-use-since-2000/), representing approximately 19 % of the average world *per capita* caloric intake and 13 % of the protein intake [1]. Italy represents the first European rice producer, with more than 50 % of the total paddy production and consumers’ requests driven by tradition and quality. Important traits influencing milling properties, appearance, grain shape, nutritional value, cooking quality and yield have recently been dissected and most of them, e.g. cooking properties, texture, gelatinization temperature (GT), chalkiness, are related to starch, seed-storage proteins (SSPs) and grain shape [2–4]. In addition, vitamin E compounds, including both tocopherols and tocotrienols, and γ-oryzanol, accumulate in the germ and in the bran fraction during grain development and are important components of rice oil, to which confer functional features thanks to their antioxidant properties [5, 6]. Eating quality therefore represents an ensemble of complex traits controlled by multiple factors [7] and to date, a number of Quantitative Trait *Loci* (QTLs) affecting rice quality have been identified [8–13]. Moreover, several genes involved in the biosynthesis and accumulation of starch, SSPs and vitamins have been characterized [3, 14, 15].

Together with amylopectin, amylose is the main component of starch and its percentage on total starch, measured as Apparent Amylose Content (AAC), represents the key determinant of rice cooking properties. High AAC cultivars (cvs), like some Italian risotto varieties, result dry and firm after cooking; whereas low AAC grains cook tender and glossy [16, 17]. The Granule-Bound Starch Synthase I (GBSS I) enzyme, encoded by the *Waxy* (*Wx*) gene, regulates starch accumulation in developing caryopsis, and the amount of amylose in grains is directly associated with the levels of the enzyme in the endosperm [18]. *Wx* is subjected also to a post-transcriptional regulation, since the presence of a Single Nucleotide Polymorphism (SNP) at the 5’ splice site of the first intron affects the pre-mRNA processing promoting alternative splicing at cryptic sites of exon 1 causing a reduced accumulation of functional enzyme and the occurrence of a glutinous phenotype [19, 20]. Other enzymes playing key roles in starch biosynthesis are ADP-Glucose Pyrophosphorylase (AGPase), which is regarded as the rate-limiting enzyme in starch biosynthesis as it catalyzes the synthesis of the starch chain primer, Starch Synthases (SS), starch Branching Enzymes (BEs) and starch Debranching Enzymes (DBEs), responsible for the biosynthesis of the branched amylopectin chains [21]. Furthermore, functional SNPs in the *SSIIa* gene are responsible for high GTs [22, 23].

Hundreds of genes encode for SSPs which not only provide a source of amino acids to be used during germination and seedling growth, but also have a deep impact on nutritional quality and food processing [24]. In rice, SSPs account for 10 % of grain composition with glutelins (Glus; counting for up to 80 % of the total SSPs) and prolamins (PROLMs) representing the main classes. Fifteen rice genes, per haploid genome, are known to encode for Glus and are classified into four subfamilies: GluA – four members, GluB – eight members, GluC – two members and GluD – one member [4]. A multigene family, composed by 80–100 copies per haploid genome, encodes instead for PROLMs [3]. Another small fraction of cereal SSPs is represented by globulins and albumins which are associated with allergenicity [25, 26]. The SSPs nutritional value is also related to the content of essential amino acids (EAs; Asn, Asp, Lys, Met, Thr, Phe, Val, Trp, Leu, Ile and His) [4].

Rice bran oil is the product of extraction of milling by-products, namely germ or bran, and represents a source of antioxidant and disease-preventative properties thanks to the presence of phytonutrients such as γ-oryzanol, basically composed by steryl ferulates, and vitamin E compounds [27]. Most of the vitamin E biosynthetic genes (*VTE* genes) have been characterized in *A. thaliana*, enabling their identification in different species, such as rice, and leading to the characterization of the biosynthetic pathways of tocopherols and tocotrienols [28–30]. On the other hand, very little is known about the biosynthesis and accumulation of γ-oryzanol even if some genes have been demonstrated to be key regulators of sterols biosynthesis [14].

Since comparisons of quality traits among cvs grown in the same environment revealed significant variations [3], it is of great interest to understand how the *loci* regulating the accumulation of starch, SSPs and VTE compounds are expressed and coordinated during grain filling. Transcription analyses during caryopsis development have shown that the highest accumulation of mRNAs for most of the starch synthase genes occurs between 6–10 Days After Flowering (DAF), with the exception of *Wx* which showed two expression peaks at 6 and 15 DAF, respectively [3]. However, Duan and Sun (2005) [31] demonstrated that the peak levels of several genes involved in the starch biosynthetic pathway could extend to 15 DAF. The expression of Glus encoding genes usually peaked at 10 DAF [32], but some studies showed increasing expression until 30 DAF for example for *GluD-1* [33]. For one 10-kDa and three 13-kDa PROLMs the same expression pattern as Glus was observed, with the exception of additional two 13-kDa PROLMs encoding genes for which transcription increases toward seed maturation (20 DAF) and of two 16 k-Da PROLMs mRNAs that accumulated until 26–28 DAF [32].

To investigate the genetic bases underlying the qualitative differences characterizing traditional Italian rice cvs, a comparative RNA-Seq-based transcriptomic analysis of developing seeds was conducted at 14 DAF on six popular Italian varieties (Arborio, Balilla, Carnaroli, Gigante Vercelli, Vialone Nano and Volano) differing for several qualitative grain-related traits. The 14 DAF timing was chosen because a number of important traits (namely AAC, protein content and chalkiness) have been reported to show a high level of expression at, or close to, 14 DAF. Co-regulation analyses of differentially expressed genes (DEGs) showing the same expression profile in the six cvs, highlighted classes of genes specifically up-regulated for each variety, leading to a transcriptional characterization of developing seeds of the main Italian rice cvs and to the identification of candidates that might affect the peculiar grain traits of the six varieties. We aimed at finding out whether the transcription profiles at 14 DAF were able to explain the qualitative differences among the Italian rice varieties studied and our results suggest that rice quality traits can be described in details through a gene expression analysis at 14 DAF, and that the qualitative differences among the main Italian rice varieties are associated to specific gene expression profiles.

## Results

### Standard phenotypic evaluations

Considering the commercial classification reported by Suwannaporn et al. (2007) [17] for AAC, the cvs analyzed were grouped in three different AAC classes (Table 1): Carnaroli (CAR, 25.4 %) - high AAC (>25 %); Arborio (ARB, 19 %) - low AAC (12-20 %); while the other genotypes belonged to the intermediate AAC class (20–25 %), with Volano (VOL, 20.3 %) and Balilla (BAL, 22.3 %) showing lower percentages of amylose with respect to Gigante Vercelli (GV, 23.5 %) and the risotto variety Vialone Nano (VN, 22.9 %). BAL represented the cv with the lowest amount of total SSPs (4.9 %), while ARB and VN showed the highest values (6.8 % and 6.7 %, respectively) (Table 1). The “EU regulation on the common organisation of the market in rice” (Council Regulation (EC) No 1308/2013; https://www.fsai.ie/legislation/legislation_update/december_2013.html#Common_Organisation_of_the_Markets_in_Agricultural_Products) distinguishes four categories, based on grain length (L) and length/width (L/W) ratio of naked caryopsis: Long A: L >6.0 mm, L/W 2.1–3.0; Long B: L >6.0, L/W > 3.0; Medium: L 5.2-6.0, L/W < 3.0; Round: L <5.2, L/W <2.0. According to EU classification the round seed variety BAL showed the shortest (7.42 mm for paddy seeds and 5.17 mm for naked caryopsis) and slimmest (3.78 mm for paddy grain and 3.13 mm for naked caryopsis) grain. This genotype was followed by the medium seed variety VN for which we detected 8.31 mm paddy seed length and 6.33 naked seed length (Table 1). All the other cvs belonged to the long A group. Analysis of tocochromanols content revealed that VN had the lowest levels for all the different classes (28.93 mgKg^−1^ DW (dry weight)), while BAL showed the highest quantity (148.59 mgKg^−1^ DW), a behaviour ascribable to the particular high amounts of α and γ-tocotrienols. GV showed relevant levels of γ and δ-tocopherols, whereas CAR was characterized by the highest amount of α-tocopherol (Table 1). The quantities of β-tocotrienol and β- and δ-tocopherols were too low to be considered (data not shown). BAL also represents the genotype with the highest amount of γ-oryzanol (1886.3 mgKg^−1^ DW), followed by CAR and VOL (1231.5 and 1022.3 mgKg^−1^ DW, respectively), while the lowest level of this compound was detected for VN (356.2 mgKg^−1^ DW) (Table 1). CAR showed the lowest milling head yield (54.0 %), followed by VN (55.5 %), while the highest values were detected for BAL (63.0 %), VOL (59.9 %) and ARB (57.5 %) (Table 1). ARB and VN displayed intermediate GT, whereas the other cvs belonged to the low GT class (Table 1). BAL grains carried small chalkiness, with a short lateral stripe. For the other genotypes we observed a large chalkiness (Table 1) that was associated to long lateral stripes, with the exception of CAR, which displayed also a central chalkiness, and VN, differing from the others for the presence of a short chalky lateral stripe.

**Table 1:** Phenotypic evaluations performed for the six cvs

	ARB	BAL	CAR	GV	VN	VOL
AAC (%)	19.0 (±0.40)	22.3 (±0.37)	25.4 (±0.73)	23.5 (±1.37)	22.9 (±0.82)	20.3 (±0.23)
Total proteins (%)	6.8 (±0.40)	4.9 (±0.46)	6.5 (±0.27)	6.1 (±0.38)	6.7 (±0.60)	6.1 (±0.25)
Paddy seed length (mm)	10.04 (±0.44)	7.42 (±0.24)	9.85 (±0.54)	9.37 (±0.44)	8.31 (±0.29)	10.00 (±0.51)
Paddy seed width (mm)	4.32 (±0.24)	3.78 (±0.20)	3.90 (±0.18)	4.14 (±0.26)	4.09 (±0.14)	4.48 (±0.16)
Paddy seed length/width	2.32	1.96	2.52	2.26	2.03	2.23
Naked caryopsis length (mm)	7.26 (±0.26)	5.17 (±0.22)	7.29 (±0.32)	6.94 (±0.33)	6.33 (±0.40)	7.28 (±0.31)
Naked caryopsis width (mm)	3.53 (±0.17)	3.13 (±0.20)	3.31 (±0.19)	3.28 (±0.26)	3.46 (±0.33)	3.61 (±0.24)
Naked caryopsis length/width	2.06	1.65	2.20	2.12	1.83	2.02
α-tocotrienol (mgKg^−1^DW)	14.74 (±1.63)	27.04 (±0.97)	19.99 (±0.53)	15.34 (±0.22)	6.26 (±0.92)	17.03 (±1.72)
δ-tocotrienol (mgKg^−1^DW)	1.98 (±0.19)	2.85 (±0.09)	3.49 (±0.12)	4.09 (±0.19)	1.93 (±0.27)	1.89 (±0.10)
γ-tocotrienol (mgKg^−1^DW)	26.38 (±2.9)	50.08 (±1.49)	32.95 (±1.20)	38.60 (±0.80)	13.42 (±1.99)	25.84 (±2.69)
α-tocopherol (mgKg^−1^DW)	57.86 (±4.49)	65.15 (±7.9)	72.04 (±5.26)	67.17 (±3.31)	6.64 (±0.91)	50.50 (±2.73)
γ-tocopherol (mgKg^−1^DW)	5.86 (±0.72)	3.47 (±0.44)	4.85 (±0.38)	9.97 (±0.52)	0.68 (±0.13)	4.21 (±0.03)
Total tocochromanols (mgKg^−1^DW)	106.82	148.59	133.32	135.17	28.93	99.47
γ-oryzanol (mgKg^−1^DW)	868.4 (±82.3)	1886.3 (±151.3)	1231.5 (±98.2)	847.2 (±53.2)	356.2 (±55.1)	1022.3 (±125.8)
Milling head yield (%)	57.5	63.0	54.0	57.4	55.5	59.0
Alkali	4	7	7	6	5	6
Chalkiness	9	1	9	9	9	9

Values represent the averages of the biological replicates and units of measurements are reported. Standard deviations are reported in brackets. *ARB* Arborio, *BAL* Balilla, *CAR* Carnaroli, *GV* Gigante Vercelli, *VN* Vialone Nano, *VOL* Volano, *AAC* Apparent Amylose Content

### RNA-Seq analysis and identification of Differentially Expressed Genes (DEGs)

**Fig. 1:**
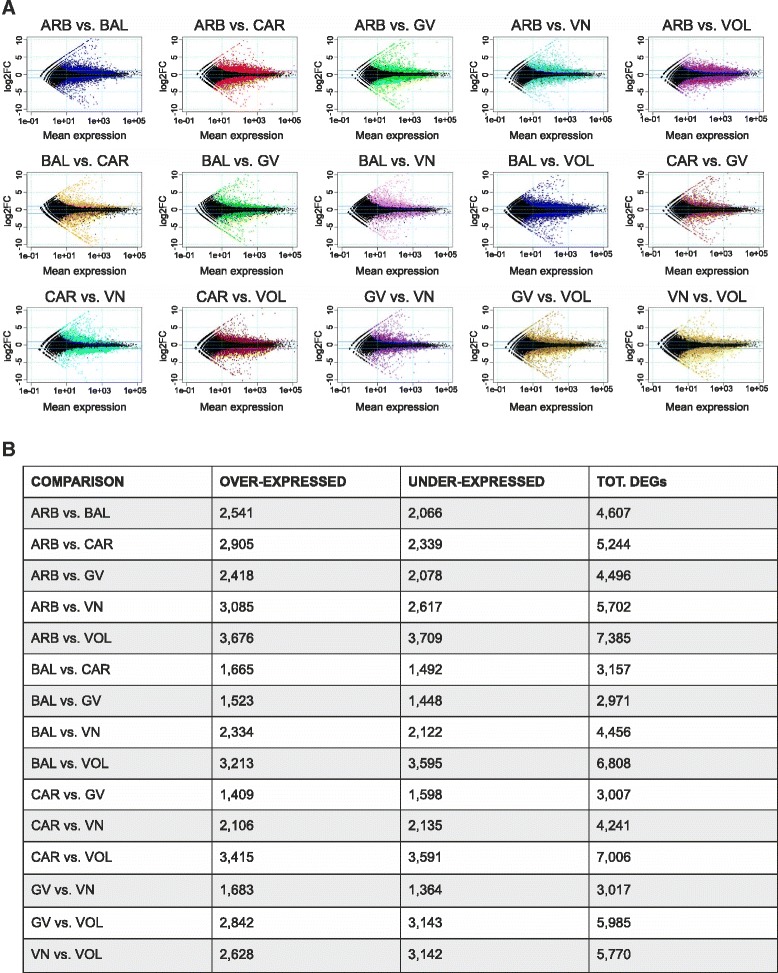
Differentially modulated genes from pair-wise comparisons. **a** Mean expression *versus* log_2_ Fold Change (FC) plots (MA-plots) representing the DEGs in the pair-wise comparisons, considering the expression in the second cv indicated with respect to the first one. Normalised expression mean values are plotted *versus* log_2_FC and DEGs are visualized as coloured dots. **b** For each comparison the numbers of over-expressed and under-expressed genes in the second cv with respect to the first one indicated and the total DEGs are reported

RNA was isolated from developing caryopsis of the six rice cvs at 14 DAF, as many genes of interest for grain quality are highly expressed in the first two weeks after fertilization, whereas others are more expressed in the subsequent weeks. In fact, 14 DAF represents a critical time-point for the determination of chalkiness and the mid-time for the biosynthesis and accumulation of starch and SSPs [2, 3, 31]. From 16 to 35 millions of filtered reads were obtained (see Additional file 1) and mapped with Bowtie/tophat to Rice Genome (*Oryza sativa* Nipponbare MSU release 6.16). An RPKM (Reads per Kilobase per Million) cutoff value of 0.1 was set to declare a *locus* expressed resulting in 31,666 *loci* above the expression cutoff in at least one genotype. Pearson correlation coefficients for biological replicates from the same genotype were always above 0.95 (see Additional file 2) indicating a good level of reproducibility (sample clustering heatmaps shown in Additional file 3). The R package DESeq was employed to normalize and compare expression data. Pair-wise comparisons among all the six genotypes were conducted to call the DEGs using a False Discovery Rate (FDR, Benjamini–Hochberg multiple test correction) threshold of 0.05. VOL was the genotype showing the highest number of DEGs when compared to the others varieties: 7385 *vs.* ARB, 7006 *vs.* CAR, 6808 *vs.* BAL, 5985 *vs.* GV and 5770 *vs.* VN. This cv showed also the highest number of under-expressed genes, with respect to the over-expressed ones, in all the comparisons. Several of the comparisons between GV and the other cvs resulted instead in the lowest numbers of DEGs: 2971 *vs.* BAL, 3007 *vs.* CAR and 3047 *vs.* VN (Fig. 1).

### Clusters analysis

**Fig. 2:**
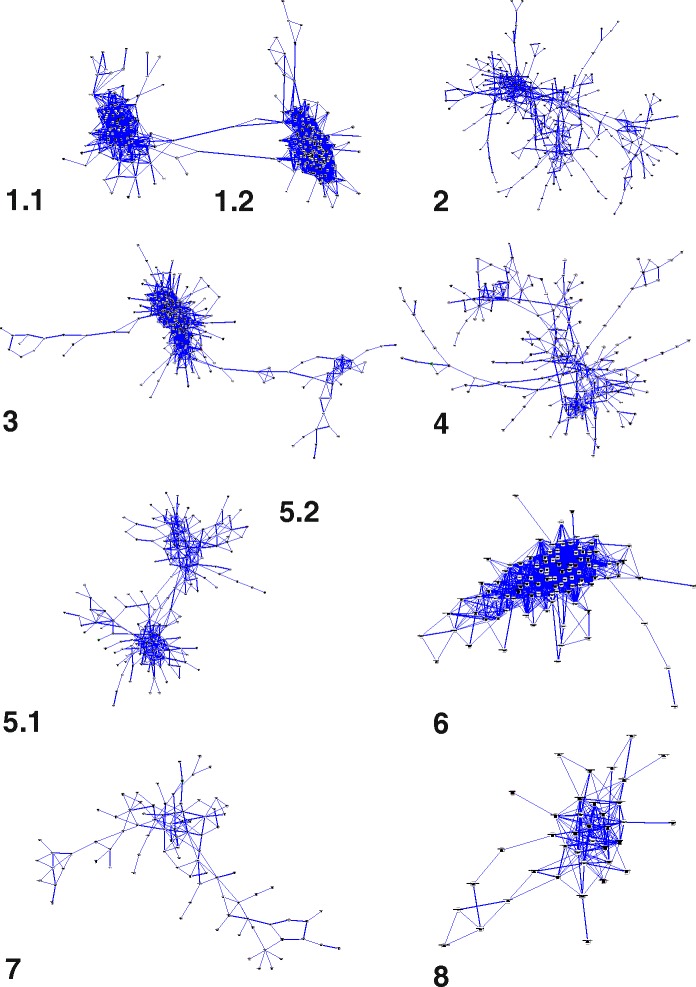
General representation of the clusters obtained by the co-regulation analysis. Numbers indicating each cluster are reported. Node colours corresponds to the mean expression values, calculated on the base of the number of reads mapping at each *locus* on rice reference genome, for all the varieties considering the three biological replicates and a scale from white, representing low expression, to purple, corresponding to high transcription

**Fig. 3:**
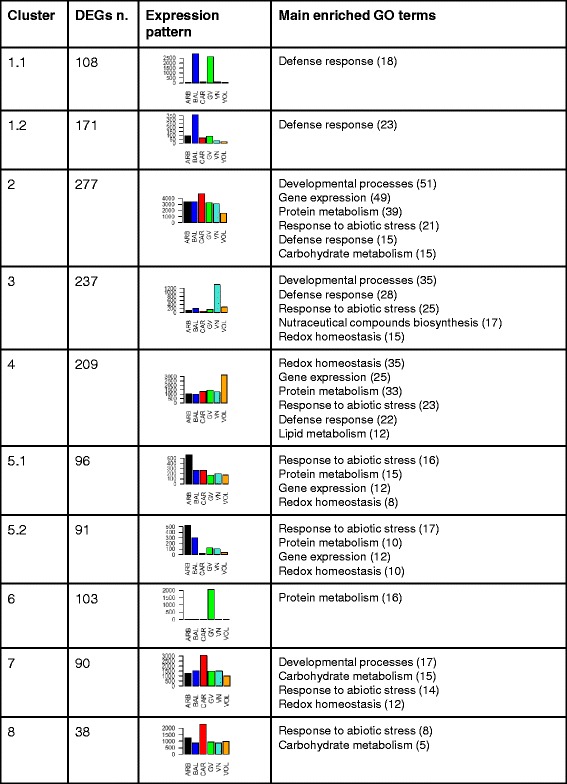
Summary of the number of DEGs detected for each cluster. Bar plots represent the expression pattern of a representative gene of the cluster in all the six genotypes. Values in bar plots correspond to the mean expression values, corresponding to the number of reads mapping at each *locus* on rice reference genome (raw read counts as obtained from DESeq-normalized matrix of expression data), of the three biological replicates (y-axis) for each cv (ARB-Arborio, BAL-Balilla, CAR-Carnaroli, GV-Gigante Vercelli, VN-Vialone Nano and VOL-Volano; x-axis). The main enriched GO terms and functional classes identified for each cluster are reported (the numbers of DEGs belonging to each class are in brackets)

In order to identify clusters of genes showing genotype-specific expression patterns, a co-expression analysis was conducted. Toward this end, a matrix of tractable size for correlation analysis was obtained by narrowing the entire gene rice pool above RPKM cutoff (31,666) to the subset of genes called as DEGs in at least one pair-wise (cv against cv) contrast and exhibiting a FC of at least 2 (10,200 genes). Eight major clusters were identified, each of them characterized by genes with a comparatively higher expression in one or at most few cvs (Fig. 2). The number of genes and the prototypical consensus expression patterns of each cluster in the six cvs are summarized in Fig. 3. The complete list of DEGs belonging to each cluster with the corresponding results for the pair-wise comparisons are indicated in Additional file 4-sheets 1 and 3, respectively. To functionally classify the members of the clusters we resorted to GO enrichment analyses and discovered peculiar enriched classes of genes in each cluster (Table 2 and Additional file 4-sheet 2).

**Table 2:** Number of DEGs in each comparison belonging to the main grain quality-related classes of genes

	Tot.	ARB vs. BAL	ARB vs. CAR	ARB vs. GV	ARB vs. VN	ARB vs. VOL	BAL vs. CAR	BAL vs. GV	BAL vs. VN	BAL vs. VOL	CAR vs. GV	CAR vs. VN	CAR vs. VOL	GV vs. VN	GV vs. VOL	VN vs. VOL
Starch biosynthesis (GO:0019252)	17 (8)	8	11	9	5	8	3	3	9	7	3	8	7	4	6	10
Starch metabolism (GO:0005982)	16 (6)	7	11	8	8	7	8	5	10	9	5	6	10	8	6	11
Starch catabolic process (GO:0005983)	10 (3)	6	6	5	7	7	7	3	3	5	4	4	6	3	3	4
Seed storage proteins (GO:0045735)	66 (17)	24	25	23	29	26	25	16	25	34	22	25	26	19	22	26
Seed storage proteins transporters	21 (14)	3	6	5	10	7	4	3	7	10	2	6	8	4	4	5
Known allergens	8 (2)	1	1	2	1	5	2	-	3	1	1	2	3	2	1	2
Essential aminoacids biosynthesis	70 (20)	9	21	11	18	28	14	10	9	15	15	18	33	12	24	17
Essential aminoacids degradation	9 (4)	-	2	3	3	5	-	-	1	4	1	-	5	1	5	2
Tocochromanols biosynthesis	27 (7)	9	13	11	12	17	6	4	2	7	3	5	12	2	11	7
Sterols biosynthesis	17 (5)	8	5	6	9	10	3	5	5	11	4	3	10	4	9	7
Grain size	8 (4)	1	4	6	2	2	5	2	1	4	4	6	5	2	4	3
Transcription factors (GO:0003700)	253 (36)	96	125	89	118	165	48	38	74	110	34	76	131	52	103	111

Total number for each category is reported and the number of DEGs putatively undergoing to alternative splicing is indicated in brackets

#### Cluster 1

Cluster 1 included two sub-clusters: cluster 1.1, consisting of 108 genes showing higher expression in both BAL and GV, and cluster 1.2, composed of 171 *loci* over-expressed only in BAL (Fig. 3 and Additional file 4). Both the sub-clusters were mainly represented by DEGs belonging to the GO term “Defence response” (GO:0006952), with 18 and 23 genes, respectively (Fig. 3 and Additional file 4-sheet 2). For example, sub-cluster 1.1 included *loci* encoding for NB-LRR resistance genes, three stripe rust resistance proteins Yr10 and the TF WRKY41, known to modulate defence signal transduction [34] (see Additional file 4-sheet 1). For sub-cluster 1.2, besides resistance genes or disease resistance proteins, we found a gene cluster on chromosome 6 encoding for thionins (THIONs), a class of proteins playing important roles in plant immunity and also of interest in human health and agribusiness [35] (see Additional file 4-sheet 1). The enrichment of defense-related genes observed in cluster 1 was not ascribable to pathogen infection, since the rice developing caryopses used for the transcriptomic analyses were not affected by pathogens. A rationale for the observed enrichment in defense-related transcripts is related to the fact that since seeds represent a rich source of food for plant pathogens, they also are important sites of plant defence. Pathogen-free seeds express genes otherwise known to be associated with pathogen infection in other tissues, presumably contributing to pre-formed or primed systems for defending the seed against pathogen attack. Activation of defense responses in pathogen-free developing seeds was indeed observed in several cereals including barley [36] and rice [37, 38].

#### Cluster 2

**Fig. 4:**
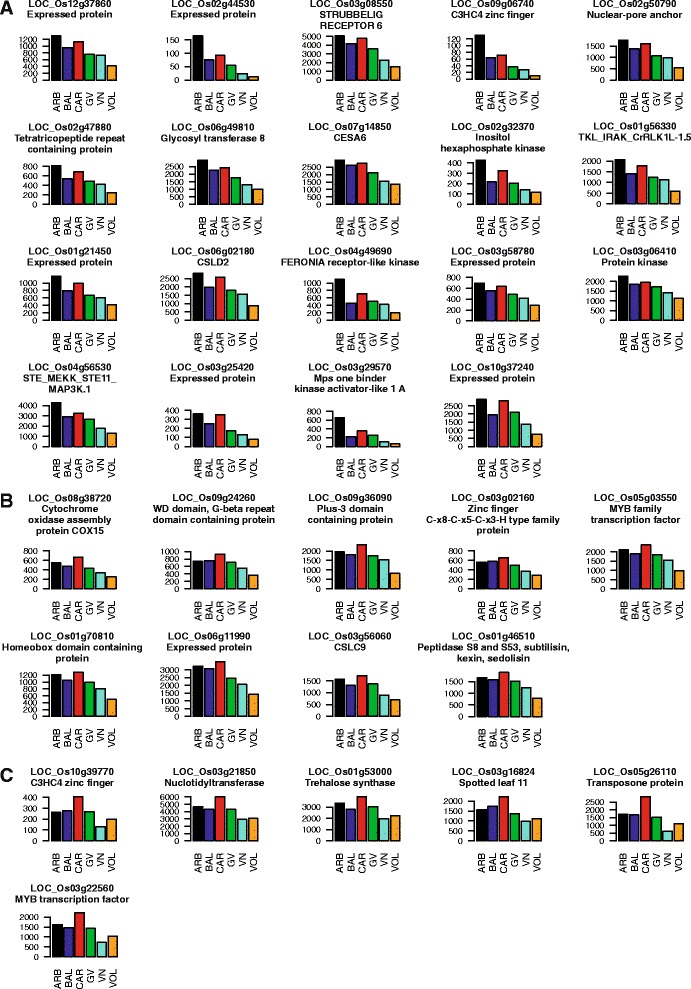
Bar-plots of some tightly co-expressed *loci* in cluster 2. **a**, **b** and **c** Different sub-clusters of tightly co-regulated *loci* belonging to cluster 2. Mean expression values, corresponding to the number of reads mapping at each *locus* on rice reference genome (raw read counts as obtained from DESeq-normalized matrix of expression data), of the three biological replicates (y-axis) for each cv (ARB – Arborio, BAL – Balilla, CAR – Carnaroli, GV – Gigante Vercelli, VN – Vialone Nano and VOL – Volano; x-axis) are represented

With 277 members, cluster 2 was the cluster containing the highest number of DEGs and showed up-regulation mainly for CAR, followed by ARB and BAL, and down-regulation for VOL. However exceptions occurred as 40 genes were mostly transcribed in ARB, followed by CAR and BAL (Fig. 3 and Additional file 4). The genes belonging to cluster 2 were mainly implicated in “Developmental process” (GO:0032502 – 51 DEGs), “Gene expression” (GO:0010467 – 49 DEGs) and “Protein metabolism” (GO:0044267 – 39 DEGs). Other well represented enriched GO terms were “Response to abiotic stimulus” (GO0009628 – 21 DEGs), “Defence response” (15 DEGs) and “Cellular carbohydrate metabolic process” (GO:0044262 – 13 DEGs) (Fig. 3 and Additional file 4-sheet 2). Three *loci* encoding for tetratricopeptide domain containing proteins, a class known to influence endosperm appearance [39], and five for proteins carrying WD domains, present in factors affecting the biosynthesis of nutraceutical compounds such as flavonoids [40], were present among the 51 DEGs affecting developmental processes (see Additional file 4-sheets 1 and 2). Among the 39 *loci* implicated in “Protein Metabolism”, 19 members belonging to “Protein phosphorylation” (GO0006468), 7 to “Protein ubiquitination” (GO:0016567), 15 to “Protein transport” (GO:0015031) and 9 chaperons were identified. This last group contained some DnaJ encoding *loci* and the DnaK-*BiP* gene (LOC_Os02g02410) which regulates the accumulation of SSPs in rice endosperm and the level of chalkiness in grains [41] (see Additional file 4-sheets 1 and 2). In the “Cellular carbohydrate metabolic process” group, genes affecting cell wall biosynthesis such as 1,3-β-glucan synthases, cellulose synthases and putative glycosyl transferases 8 were present. The glycosyl transferase 8 encoded by LOC_Os06g49810 and the cellulose synthases CESA6 (LOC_Os07g14850) and CSLD2 (LOC_Os06g02180) were tightly co-regulated with LOC_Os02g47880, encoding for a tetratricopeptide repeat domain containing protein and several *loci* involved in signal transduction: the STRUBBELIG–RECEPTOR LOC_Os03g08550, the GATA zinc finger LOC_Os02g56250, the STE_MEKK_ste11_MAP3K.1 LOC_Os04g56530, the CrRLK1L-1 LOC_Os01g56330, a FERONIA receptor kinase (LOC_Os04g49690), belonging to a class of proteins demonstrated to regulate cell wall biosynthesis [42], and a mps gene (LOC_Os03g29570) (Fig. 4a and Additional file 4-sheet 3). Different TFs were identified in the “Gene expression” GO term, including MYB TFs and zinc fingers of types C-x8-C-x5-C-x3-H, C3HC4, C2H2, CW and GATA. Moreover, two TFs positively affecting rice grain length, *OsWRKY78* - LOC_Os07g39480 and the Apetala 2 (AP2) TF *SRS1* - LOC_Os07g42410 [43, 44], were detected (see Additional file 4-sheets 1 and 2). Interestingly, through the homeobox *locus* LOC_Os01g70810, the cellulose synthase CSLC9 (LOC_Os03g56060) was tightly associated to the MYB LOC_Os05g03550 and to the C-x8-C-x5-C-x3-H zinc finger LOC_Os03g02160 (Fig. 4b and Additional file 4-sheet 3); while the MYB LOC_Os03g22560 belonged to a sub-cluster including LOC_Os10g39770 (C3HC4 type zinc finger) and LOC_Os01g53000 (trehalose synthase) (Fig. 4c and Additional file 4-sheet 3). Other functional groups for cluster 2 were represented by 6 genes acting in “Lipid metabolism” (GO:0006629), four of which affecting wax biosynthesis, 9 DEGs involved in the biosynthesis of nutraceutical compounds, like flavonoids and arabinogalactans, and 11 *loci* implicated in “Oxidation-reduction process” (GO:0055114) (see Additional file 4-sheets 1 and 2).

#### Cluster 3

Cluster 3 was represented by 237 genes mostly expressed in VN (Fig. 3 and Additional file 4). With respect to the other clusters, cluster 3 showed the highest representation of the GO terms “Defense response” (28 members) and “Response to abiotic stimulus” (GO:0009628; 25 members). In this last class, genes implicated in “Response to radiation” (GO:0009314) and “Response to wounding” (GO:0009611) were identified. Seventeen cluster 3-specific DEGs encoded for proteins involved in the biosynthesis of nutraceutical compounds, such as alkaloids (berberine and reticuline oxidase-like protein precursor), flavonoids (calcone synthase), terpenoids (cytochrome P450), glycans (alpha-1,4-fucosyltransferase) and glucans (exostosin family domain containing protein). However, seven *loci* corresponded to allergenic proteins: LOC_Os01g74480 - cupin domain containing protein, LOC_Os05g10830 - proteophosphoglycan ppg4, LOC_Os12g43490 - thaumatin and four LTP (Lipid Transfer Protein) allergens. Other well represented GO terms for cluster 3 were “Developmental process”, containing 35 *loci*, and “Oxidation-reduction process” (GO:0055114), including a total of 15 DEGs, 5 of which encoding for cytochromes P450, 3 for glutathione S-transferases, 3 for oxidoreductases and 2 for peroxidases (see Additional file 4-sheets 1 and 2).

#### Cluster 4

Two hundred nine genes up-regulated in VOL belonged to cluster 4 (Fig. 3 and Additional file 4) which showed the highest number of DEGs (35) implicated in “Oxidation-reduction process”, including 6 *loci* encoding for cytochromes and 4 for glutathione S-transferases, and for “Lipid metabolism” (GO:0006629). Another enriched GO term well represented in this cluster was “Protein metabolism”, counting for 33 *loci*, with both the GO terms “Protein transport” (GO:0015031) and “Proteolysis involved in cellular protein catabolic process” (GO:0051603) including 6 genes (see Additional file 4-sheet 2). Moreover, different *loci* implicated in protein biosynthesis, like the ribosomal proteins S26, S29, S9-2, L37, L5 and S17, and the glutamine synthetase LOC_Os03g50490 were identified. Besides these, 5 genes participated to nutraceutical compounds biosynthetic pathways, like the palmitoyl-protein thioesterase 1 precursor (LOC_Os03g01150) and the terpene synthase (LOC_Os03g24760), involved in the biosynthesis of γ-oryzanol (Piironen et al., 2000). However, the precursor of RA5/RA14/RA17 allergens RAL6 (LOC_Os07g11510), which accumulates 15–20 days after flowering [45], belonged to this cluster (see Additional file 4-sheet 1).

#### Cluster 5

Cluster 5 consisted of two sub-clusters: 5.1, containing 96 DEGs more expressed in ARB with respect to all the other cvs, and 5.2, including 91 DEGs up-regulated in ARB, followed by BAL, GV and VN (Fig. 3 and Additional file 4). The most enriched GO classes for both the sub-clusters were “Response to abiotic stimulus” (16 and 17 DEGs for 5.1 and 5.2, respectively) and “Protein metabolism” (15 and 10 DEGs, respectively). For this last group, factors involved in protein phosphorylation, ubiquitination and folding were identified (see Additional file 4-sheet 2). Moreover, 12 DEGs affecting gene expression were detected for both the sub-clusters which included TFs such as GATA zinc finger, bZIP, OsMADS87 and OsWRKY35. *Loci* affecting cell wall biosynthesis were specifically identified for sub-cluster 5.1 and included the cellulose synthase CESA9, a polygalacturonase inhibitor, a xylanase inhibitor and the NAC domain-containing protein 67, belonging to a class of TFs which activate the biosynthesis of cell wall [46]. Even with only two members, the thiamine biosynthesis protein thiC LOC_Os03g47610 and the FAD dependent oxidoreductase domain containing protein LOC_Os07g34570, the GO term “Vitamin biosynthetic process” (GO:0009110) was a prerogative of sub-cluster 5.2 (see Additional file 4-sheet 2).

#### Cluster 6

Cluster 6 was represented by 103 DEGs up-regulated in GV and mainly enriched of *loci* implicated in protein metabolism (16) and in particular in protein phosphorylation (10) (Fig. 3 and Additional file 4-sheet 2). One Glu encoding gene (LOC_Os02g16830) and three PROLMs corresponding *loci* (PROLM27 - LOC_Os12g16880, PROLM29 - LOC_Os12g17010 and PROLM30 - LOC_Os12g17030) were also identified as belonging to this cluster (see Additional file 4-sheet 1).

#### Cluster 7

Cluster 7 included 90 genes over-expressed in CAR, with some exceptions showing higher expression in BAL, followed by CAR (Fig. 3 and Additional file 4). With, 15 DEGs, cluster 7 showed the highest enrichment for the GO term “Carbohydrate metabolism” which included a SS, an amidase, a glucan endo-1,3-beta-glucosidase, the Os5bglu19 - beta-glucosidase and some genes involved in glycolytic processes. Moreover, inside this GO term, DEGs affecting the “Secondary cell wall biogenesis” were also detected. Examples are LOC_Os03g30250, encoding for a COBRA-like protein precursor, belonging to a class of proteins regulating cellulose deposition [47], and four *loci* encoding for proteins carrying a DUF domain, which has been hypothesized to have a role in cell wall biosynthesis [48]. Also 7 *loci* implicated in nutraceutical compounds metabolism like arabinogalactans (LOC_Os01g47780 and LOC_Os03g57460) and thiamines (LOC_Os05g33840; GO:0009228) were identified in this cluster. Furthermore, genes encoding for known allergens like LTPL65 (LOC_Os01g59870) and LTPL83 (LOC_Os03g58940) were identified. Three DEGs corresponded to enzymes involved in lipid biosynthesis and, in particular, LOC_Os03g12030 encoded for a 3-ketoacyl-CoA synthase belonging also to the GO term “Wax biosynthesis” (GO:0010025), together with other four genes (see Additional file 4-sheets 1 and 2).

#### Cluster 8

Cluster 8 included 38 CAR up-regulated genes (Fig. 3 and Additional file 4), eight of which involved in the response to abiotic stresses and five in cell wall biosynthesis, like the chitinase CHIT6 (LOC_Os05g33150) and the glucan endo-1,3-β -glucosidase-like protein 3 precursor encoded by LOC_Os05g43690.

### Genes affecting grain quality

To analyse the expression profiles of other genes affecting grain quality, not belonging to the main co-expression clusters, we performed GO enrichment analyses of all the DEGs identified in the pair-wise comparisons. A total of 17, 16 and 10 DEGs for “Starch biosynthetic process” (GO:0019252), “Starch metabolism” (GO:0005982) and “Starch catabolic process” (GO:0005983), respectively, were identified. In addition, 66 DEGs were detected for “SSPs” (GO:0045735), while 253 were discovered for “TFs” (GO:0003700) (Table 2). Since a GO classification for SSPs transporters, allergens, regulation of seed size, EAs biosynthesis and degradation, tocochromanols and sterols biosynthesis (Table 2) was not available, literature searches implemented with the use of the Gramene database (http://www.gramene.org) and the MAPMAN software [49], were applied for these categories, leading to the detection of 21, 8, 8, 70, 9, 27 and 17 total DEGs, respectively for each of the category indicated (Table 2).

#### Starch

**Fig. 5:**
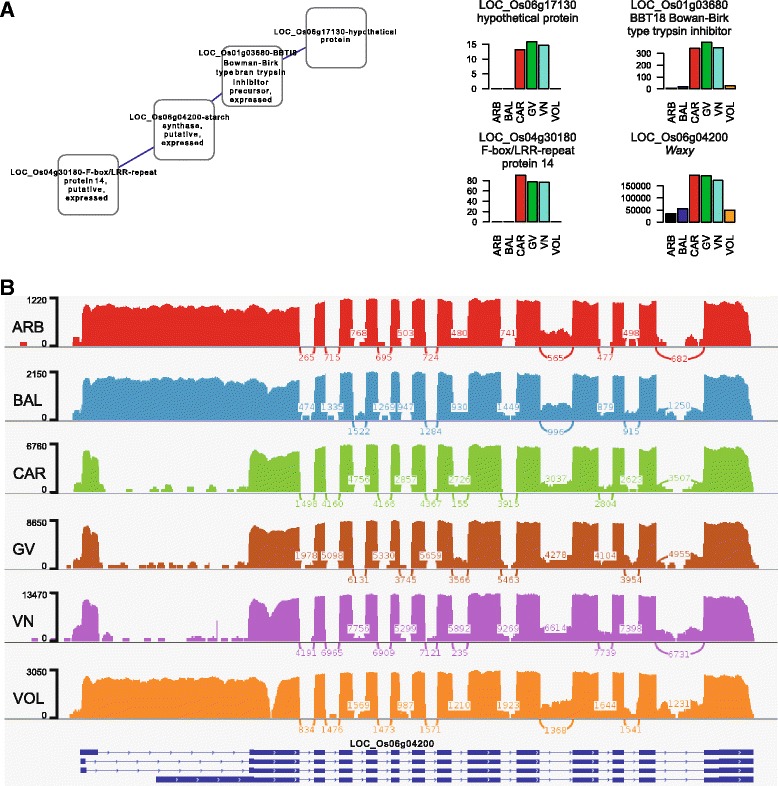
Expression of genes belonging to the *Wx* cluster. **a** Representation of the *Wx* cluster and Bar-plots indicating the mean expression values, corresponding to the number of reads mapping at each *locus* on rice reference genome (raw read counts as obtained from DESeq-normalized matrix of expression data), of the three biological replicates (y-axis) for each cv (ARB–Arborio, BAL–Balilla, CAR–Carnaroli, GV–Gigante Vercelli, VN–Vialone Nano and VOL–Volano; x-axis). **b** Sashimi plots of RNA-Seq reads aligned at LOC_Os06g04200, on Nipponbare Reference Genome, in the six different varieties. Only one biological replicate for each genotype is represented. The alignments were performed using the IGV software. The coverage for each alignment track is plotted as a bar graph. Coverage ranges for each variety are reported in the bars on the left. Arcs represent splice junctions. Junction depth is indicated by the number in the arc and the thickness of the arc. Graphs were built setting a Min Junction Coverage corresponding to the 1 % of the mean exons coverage. The alternative isoforms of the gene annotated in the Rice Genome browser MSU database are reported in the lower bar. Thick and intermediate boxes represent exons and 5’ and 3’-UTR regions, respectively. Lines between boxes indicate introns. The mapping coverage for each variety at the level of each exon and intron is represented by the coloured parts

**Fig. 6:**
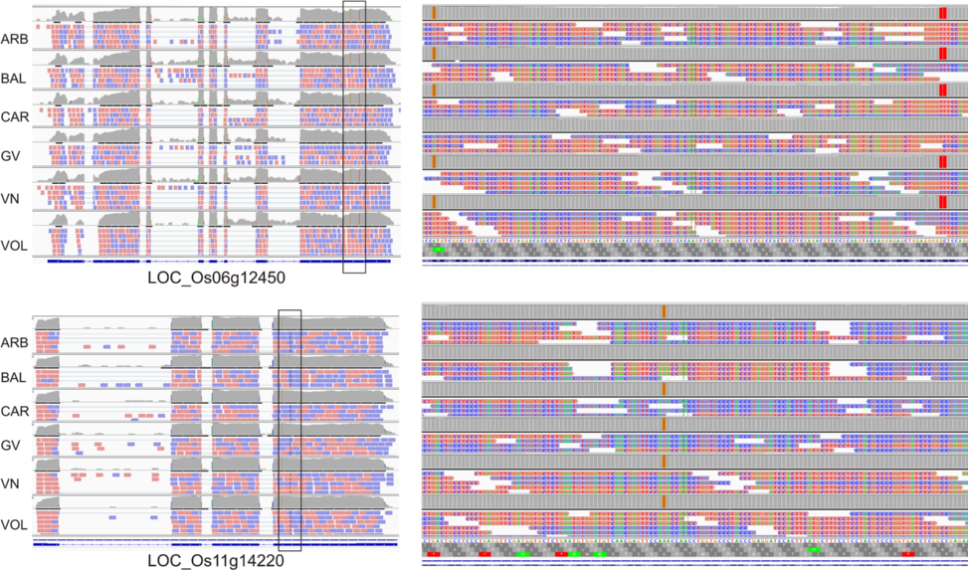
IGV outputs of the alignments of the RNA-Seq reads for each cv at the LOC_Os06g12450 and LOC_Os11g14220 *loci* on Nipponbare Reference Genome (left part). Only one biological replicate for each genotype is represented. The structures of the genes annotated in the Rice Genome browser MSU database are reported in the lower bar. Thick and intermediate boxes represent exons and 5’ and 3’-UTR regions, respectively. Lines between boxes indicate introns. The mapping coverage for each variety at the level of each exon and intron is represented by the upper grey parts. Forwards reads are in red, reverse reads are in blue. Enlargement of the sections indicated by black squares are represented on the right part of the Figure. Putative functional SNPs are indicated as coloured bars: orange for G to A and red for G to T and C to T

The highest number of DEGs belonging to starch biosynthesis and metabolism were detected in the comparisons ARB *vs.* CAR, VN *vs.* VOL and BAL *vs.* VN, while the starch catabolic process GO term was more enriched in ARB *vs.* VN, ARB *vs.* VOL and BAL *vs.* CAR (Table 2). As expected, the *Waxy* gene (LOC_Os06g04200) was more expressed (from 3 to 5 times) in the varieties showing the highest values of AAC (CAR, GV and VN) with respect to the other cvs (Fig. 5a and Additional file 5). This gene belongs to a small co-regulated cluster of four *loci*, including LOC_Os06g17130 corresponding to a hypothetical protein, LOC_Os04g30180, encoding for a F-box/LRR-repeat protein 14, belonging to a class of proteins involved in protein ubiquitination [50], and LOC_Os01g03680, corresponding to a Bowman-Birk type bran trypsin inhibitor precursor - BBTI8, belonging to a group of factors with antioxidant capacity [51] (Fig. 5a). IGV (Integrative Genome Viewer) visualization of the reads mapping at the *Wx locus*, on the Nipponbare Reference Genome, for the six genotypes, revealed the retention of the first intron, typical of the *Wx*^*b*^ allele, for the low AAC ARB, BAL and VOL (Fig. 5b), in all the three biological replicates (data not shown). On the other hand, the high AAC genotypes had a complete splicing (*Wx*^*a*^ allele) (Fig. 5b). PCA analyses conducted using the expression values for the six cvs of all the DEGs involved in starch biosynthesis, catabolism and metabolism, showed that 8.82 % of phenotypic variance (component 2: PC2; see Additional file 6) was due to the expression of a single gene identified in *Wx*. AAC is also influenced by the ADP-glucose pyrophosphorylase (AGPase), the first starch biosynthetic enzyme in seeds [52]. The seed-specific isoform of the small subunit of AGPase (*OsAGPS2*; LOC_Os08g25734) was from 0.3 to 0.57 less expressed in the round seed BAL with respect to the high AAC varieties (see Additional file 5). Conversely, LOC_Os05g50380 encoding for the regulative large subunit of AGPase, positively linked to grain weight [53], was more expressed in the longest seed VOL, but less transcribed in the medium grain VN (see Additional file 5). Despite the fact that the main determinant of GT, SSIIa (LOC_Os06g12450), was not highly differentially expressed in the pair-wise comparisons (see Additional file 5), two alleles, both defined by non synonymous SNPs, were identified in all the three biological replicates among our genotypes performing IGV mapping at the *SSIIa locus* (Fig. 6). GV carried the GTG to ATG SNP, with respect to the Nipponbare reference sequence, at 4198 bp, while the other cvs showed the CTC to TTC mutation at 4330 bp. Both the mutations corresponded to characterized functional SNPs [4] (Fig. 6). The only covalent modification of starch is phosphorylation, catalized by the α-Glucan Water Dikinase (GWD) enzyme (LOC_Os06g30310) [54] which showed lower expression, with FC values ranging from 1.5 to 3, in the main Italian risotto varieties CAR and VN, with respect to the other cvs (see Additional file 5).

#### Seed storage proteins and allergens

BAL *vs.* VOL showed the highest number of DEGs for the “SSPs” GO term (34), while BAL *vs.* GV and GV *vs.* VN showed the lowest (16 and 19, respectively). Among the 65 DEGs belonging to this GO term, 9 encoded for PROLMs, 13 for Glus and 3 for albumins (Table 2 and Additional file 5). BAL, carrying the lowest amount of protein in grains (4.9 %; Table 1), showed the lowest level of transcription for most of the Glus encoding *loci*. Conversely, the highest transcription for Glus was detected for CAR (6.5 % of protein content; Table 1). ARB is the genotype with the highest amount of proteins (6.8 %; Table 1) and showed higher expression of the Glu *locus* LOC_Os02g25860, with respect to all the other genotypes. Despite the fact that VN is the second cv with the highest level of proteins in seeds (6.7 %; Table 1), it did not show particular over-expression of genes encoding for Glus. In this group, seven genes (LOC_Os02g15090, LOC_Os03g31360, LOC_Os02g25640, LOC_Os02g15150, LOC_Os10g26060, LOC_Os01g55690 and LOC_Os02g16820) were predicted to be subjected to alternative splicing (Table 2 and Additional file 5). GV and VOL, having both 6.1 % of proteins in seeds, represented the genotypes with the highest expression of PROLMs. As mentioned above, PROLM27, PROLM29 and PROLM30 belonged to the GV up-regulated cluster 6, while VOL, with FCs ranging from about 2 to about 4, showed the highest expression for PROLM25 (LOC_Os07g10570) and PROLM26 (LOC_Os07g10580). For both the cvs, high transcription was also observed for PROLM20 (LOC_Os07g11910) and PROLM19 (LOC_Os07g11900). No alternative splicing was predicted for this class of genes (see Additional file 5). The seed specific RISBZ1 (LOC_Os07g08420) bZIP TF, activating the transcription of SSPs encoding genes in rice [55], was about two times more expressed in VOL with respect to the other cvs (see Additional file 5). Several genes known to regulate the deposition of SSPs into Protein Bodies (PBs) were identified among DEGs (see Additional file 5). These included the BiP chaperon (LOC_Os02g02410), slightly (almost two times) more expressed in CAR as mentioned above, Rab5 GTPase up-regulated in VN (LOC_Os05g01994), three genes encoding for different isoforms of Sar1 GTPase (LOC_Os01g23620-Sar1a, LOC_Os12g37360-Sar1b and LOC_Os12g37360-Sar1c), and RBP (RNA Binding Protein), involved in the accumulation of PROLMs, with the highest FC values (from about 2 to about 5) identified for RBP-B (cp29 - LOC_Os07g25960), mostly expressed in VOL (see Additional files 4-sheet 3 and 5). Analyzing *loci* belonging to the co-regulation clusters, we could identify genes encoding for allergenic proteins specifically more expressed in certain varieties, with respect to the others. The expression profiles in the pair-wise comparisons of other genes belonging to this class, but not included in the co-regulated clusters, was also dissected. In addition to the DEGs in the up-regulated clusters, VOL and VN displayed over-expression also for different LTPLs, including LTPL8 (LOC_Os11g02400) for both the genotypes, and LTPL13 (LOC_Os12g02330), LTPL32 (LOC_Os11g14880) for VOL. Furthermore, CAR-specific up-regulation, with respect to the other cvs, was detected for LTPL7 (LOC_Os11g02369) and LTPL11 (LOC_Os12g02310). Conversely, BAL showed the lowest transcription rates for LTPL124 (LOC_Os04g52260) (see Additional file 5). One of the major allergenic classes of proteins in rice is represented by albumins [56] and, among our DEGs, three *loci* (LOC_Os03g55740, LOC_Os11g33000 and LOC_Os05g41970) encoded for precursors of 2S albumins, with significant differences in expression in most of the pair-wise comparisons. Down-regulation was observed for BAL, while CAR and VOL displayed double expression for LOC_Os11g33000 and LOC_Os05g41970, respectively, in comparison to the other accessions (see Additional file 5). Other typical allergenic SSPs are the Cupin domain containing proteins [57] for which we identified 15 encoding DEGs. In general, BAL and ARB had the lowest expression, while VN showed the highest (see Additional file 5). As examples, for LOC_Os08g09040 the expression in VN was about 13, 36 and 8 times higher with respect to ARB, BAL and CAR, respectively, while for LOC_Os01g74480 the FCs were about 18, 9 and 14 in comparison to BAL, CAR and GV, respectively (see Additional file 5).

#### Nutraceutical compounds

Analysis of the pair-wise comparisons data focused on genes associated to nutraceutical compounds highlighted that VOL showed up-regulation for two genes involved in terpenoids biosynthesis and encoding for the terpene synthase LOC_Os04g52210 and the ent-kaurene synthase LOC_Os04g52230. Moreover, a small cluster of five genes containing a flavonol synthase (LOC_Os05g03640) tightly associated to a DUF domain containing protein (LOC_Os02g52930) in turn related to a MYB TF (LOC_Os03g55590), belonging to a class of TF that regulates flavonol biosynthesis in rice [48], showed higher expression in CAR (see Additional file 5).

According to Gramene database annotation, a total of 70 and 9 DEGs were identified as involved in EAs biosynthesis and catabolism, respectively. Among these, 12 and 5, respectively, were predicted to be subjected to alternative splicing (Table 2). On the bases of our comparisons, a peculiar regulation of genes belonging to this category for each genotype was highlighted. VOL represents the cv with the highest number of modulated genes, with 10 and 3 DEGs involved in the biosynthesis and degradation of EAs, respectively, and particularly belonging to the Asp family. Furthermore, ARB displayed down-regulation of two genes involved in Asp biosynthesis and two genes belonging to the Trp biosynthetic pathway. Two *loci* participating to His biosynthesis were specifically more transcribed in GV (see Additional file 5).

A total of 27 unique genes involved in tocochromanols biosynthesis were identified as DEGs in our experiment (Table 2 and Additional file 5). ARB was the genotype with the highest number of modulated genes in most of the comparisons: about 6, all down-regulated. By contrast, CAR showed up-regulation for several *loci*. Another nutraceutical component of rice brain oil is γ-oryzanol which is composed by ferulic acid esters of sterols and triterpene alcohols [6]. Very little is known about the biosynthesis of γ-oryzanol, even if some pivotal genes involved in the phytosterols biosynthetic pathways have been characterized [14]. Among these, 17 were identified as DEGs in our comparisons (Table 2). As a general trend, VOL had the highest number of modulated genes for this group: 6 and 2, up and down-regulated, respectively, in most of the pair-wise comparisons (see Additional file 5).

#### Grain size

In addition to the genes affecting grain biometry described above in the cluster session, eight DEGs identified in the pair-wise comparisons belonged to this category (Table 2 and Additional file 5). The negative regulator of grain width *GW2* (LOC_Os02g14720) [58] was about two times more expressed in CAR and GV with respect to the other cvs (see Additional file 5). Moreover, CAR had double expression in most of the comparisons for *D61* (LOC_Os01g52050), encoding for a BR (brassinosteroid) insensitive 1 (BRI)-like leucine-rich repeat (LRR) receptor kinase regulating seed size affecting brassinosteroid biosynthesis [59]. The *GIF1* gene (LOC_Os04g33740), corresponding to a cell-wall invertase required for carbon partitioning during grain filling which positively affects grain dimensions [44], was two times more expressed in GV, with respect to all the other accessions, and about 2 times less transcribed in the medium cv VN in comparison to the long A genotypes. No large variation in expression levels was detected, in our pair-wise comparisons, for the *SRS5* gene (LOC_Os11g14220) which encodes for an α-tubulin that regulates cell elongation [60] (see Additional file 5). However, the IGV mapping to the Nipponbare Reference Genome underlined that the round seed BAL carried a single SNP in all the reads mapping at that *locus*, with respect to the other genotypes (Fig. 6), for all the three biological replicates (data not shown). This mutation corresponds to the polymorphism in exon 4 known to be responsible for a small seed size [60]. Four of the DEGs involved in grain size (LOC_Os02g14720, LOC_Os04g33740, LOC_Os11g14220 and LOC_Os07g42410) were predicted to undergo to alternative splicing (Table 2 and Additional file 5).

#### Transcription factors

In the TFs GO term (GO:0003700) a total of 253 DEGs were identified, thus representing the richest class of modulated genes in our experiment, with ARB *vs.* VOL showing the highest number of *loci* (165) and CAR *vs.* GV the lowest (34) (Table 2 and Additional file 5). The most representative TF families identified were the WRKY superfamily, accounting for 25 DEGs, and the AP2 superfamily with 40 DEGs (see Additional file 5). VN was the genotype displaying an over-all higher expression of WRKY TFs in the pair-wise comparisons with FC ranging from about 2 to about 37, with respect the other genotypes. Examples are LOC_Os01g61080 (*OsWRKY24*), LOC_Os05g25770 (*OsWRKY45*) and LOC_Os05g27730 (*WRKY53*) (see Additional file 5). In general, ARB and BAL showed the highest expression for the AP2 class; by contrast, down-regulation was detected mainly for VOL (see Additional file 5). Among the other TFs encoding DEGs, 20 belonged to the MADS-box family, 40 were homeobox genes, 7 of which carried a START domain, 19 contained a bZIP domain and 21 corresponded to zinc finger domain containing proteins (12 GATA, 3 C2H2, 1 CCT/B-box, 1 NF-X1-type and 1 Dof) (see Additional file 5).

### Time course analysis of selected genes by quantitative real-time PCR

**Fig. 7:**
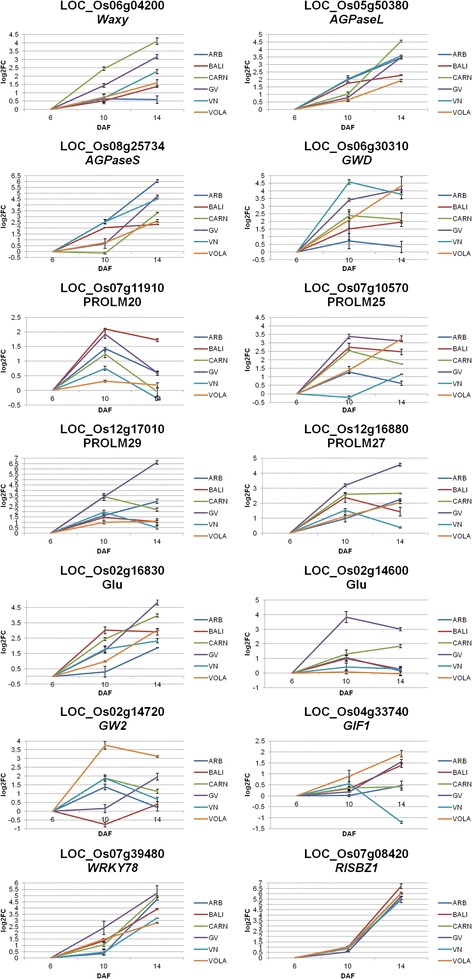
qRT-PCR at 6, 10 and 14 DAF for the 14 selected DEGs in all the six varieties. Values are expressed as log_2_FC of expression at 10 and 14 DAF in comparison to 6 DAF, respectively. FCs are expressed as mean of the three biological replicates. Bars indicate standard errors across for the three biological replicates considering the three technical replicates for each of them

Gene expression analyses, by means of quantitative real-time PCR (qRT-PCR), were conducted at 3 time points (6, 10 and 14 DAF) for a total of 14 *loci* belonging to the main classes affecting grain quality identified: starch metabolism (LOC_Os06g04200-*Wx*, LOC_Os05g50380-*AGPase* large subunit, LOC_Os08g25734-*AGPase* small subunit and LOC_Os06g30310-*GWD*), SSPs (LOC_Os07g11910-PROLM20, LOC_Os05g10570-PROLM25, LOC_Os12g17010-PROLM29, LOC_Os12g16880-PROLM27, the two Glus encoding LOC_Os02g16830 and LOC_Os02g14600 and LOC_Os07g08420-RISBZ1) and grain size (LOC_Os02g14720-*GW2*, LOC_Os04g33740-*GIF1* and LOC_Os07g39480-*WRKY78*). Rice Ubiquitin conjugating enzyme E2 (AK059694) was used as the housekeeping gene. Reactions were performed for the three biological replicates for each cv, with three technical replicates each, for all the time-points and results were reported as mean values of the three biological replicates. As control, the same primer pairs utilized for qRT-PCRs were used to amplify the genes from genomic DNA of the six cvs (data not shown). The comparison between RNA-Seq data and qRT-PCRs at 14 DAF resulted in a substantial agreement for the differential expression of the genes in the six cvs (Table 3), validating the RNA-Seq analysis. The time course expression analysis was performed comparing the transcription levels at 10 and 14 DAF with those at 6 DAF (Fig. 7). The *Wx* gene and the two subunits of AGPase resulted more expressed at both 10 and 14 DAF with respect to 6 DAF, with the highest mRNA levels at 14 DAF for all the cvs excluding the small and round seed BAL which showed the highest expression at 10 DAF (Fig. 7 and Additional file 7). Moreover, pair-wise comparisons revealed that the high AAC genotypes (CAR, GV and VN) had higher expression for *Wx*, with respect to the low AAC even at 6 and 10 DAF (see Additional file 7). The time course expression analyses of the *GWD* gene (LOC_Os06g30310) revealed differences among the six varieties: no expression changes were observed at the time-points analyzed for ARB, while an increase of the transcription rate was observed from 6 to the later maturation stages for GV, VOL and BAL and a strong decreased expression at 14 DAF was observed for the two Italian risotto varieties CAR and VN (Fig. 7 and Additional file 7). Also for the SSPs encoding genes, large variations were observed, with LOC_Os02g16830 showing an increasing expression until 14 DAF and LOC_Os07g11910 peaking at 10 DAF. For the other SSPs *loci* a constant increasing of transcription towards maturation was detected for GV and ARB at LOC_Os12g17010, for GV, ARB and VOL at LOC_Os12g16880 and for VOL and VN at LOC_Os07g10570 (Fig. 7). Both RISBZ1 (LOC_Os07g08420) and *OsWRKY78* (LOC_Os07g39480) peaked at 14 DAF for all the cvs (Fig. 7). Moreover, LOC_Os07g39480 was about two times more expressed in ARB with respect to the other long A genotypes (CAR, GV and VOL) at 6 DAF (see Additional file 7). A cv-specific time dependent regulation for LOC_Os02g14720 (*GW2*) and LOC_Os04g33740 (*GIF1*) was observed. For LOC_Os02g14720 a peak of expression at 14 DAF was observed in BAL and GV, while higher transcription at 10 DAF was detected for the other genotypes. For LOC_Os04g33740, all the cvs showed highest mRNA levels at 14 DAF, excluding VN that displayed down-regulation at 14 DAF (Fig. 7).

**Table 3:** Comparison of qRT-PCR FCs in the pair-wise comparisons at 14 DAF and RNA-Seq FCs

	ARB/BAL		ARB/CAR		ARB/GV		ARB/VN		ARB/VOL		BAL/CAR		BAL/GV		BAL/VN		BAL/VOL		CAR/GV		CAR/VN		CAR/VOL		GV/VN		GV/VOL		VN/VOL	
LOCUS	qRT-PCR	RNA-Seq	qRT-PCR	RNA-Seq	qRT-PCR	RNA-Seq	qRT-PCR	RNA-Seq	qRT-PCR	RNA-Seq	qRT-PCR	RNA-Seq	qRT-PCR	RNA-Seq	qRT-PCR	RNA-Seq	qRT-PCR	RNA-Seq	qRT-PCR	RNA-Seq	qRT-PCR	RNA-Seq	qRT-PCR	RNA-Seq	qRT-PCR	RNA-Seq	qRT-PCR	RNA-Seq	qRT-PCR	RNA-Seq
LOC_Os06g04200 *GBSSI*	0.97	0.67	3.68	2.48	3.41	2.46	3.04	2.31	0.64	0.53	2.71	1.82	2.44	1.79	2.07	1.65	−0.33	ND	−0.27	ND	−0.64	ND	−3.04	−1.96	−0.38	ND	−2.77	−1.94	−2.40	−1.79
LOC_Os06g30310 *GWD*	−0.04	ND	−0.55	−0.92	0.80	ND	−0.48	ND	0.79	0.64	−0.51	−0.64	0.84	0.62	−0.44	ND	0.82	0.92	1.35	1.27	0.07	0.56	1.33	1.56	−1.28	−0.71	−0.01	ND	1.27	1.00
LOC_Os05g50380 *AGPaseL*	1.03	0.72	0.25	ND	0.83	0.47	0.31	ND	1.83	1.15	0.17	ND	0.21	ND	−1.66	−0.78	0.75	0.44	0.12	ND	−0.88	−0.58	1.12	0.63	−0.32	−0.53	1.13	0.69	1.75	1.22
LOC_Os08g25734 *AGPaseS*	1.42	1.46	0.80	0.66	0.77	0.58	−0.35	ND	0.52	0.79	−0.62	−0.80	−0.64	−0.87	−1.77	−1.59	−0.90	−0.66	−0.02	ND	−1.15	−0.79	−0.28	ND	−1.13	−0.71	−0.26	ND	0.87	0.92
LOC_Os07g11910 PROLM20	0.53	ND	−1.58	−8.57	0.15	ND	−1.34	−9.94	0.36	ND	−2.11	−8.37	−0.39	ND	−1.88	−9.75	−0.18	ND	1.72	9.08	0.23	ND	1.93	8.96	−1.49	−10.45	0.21	ND	1.70	10.33
LOC_Os07g10570 PROLM25	0.04	ND	−0.09	ND	0.62	ND	−0.07	ND	2.74	1.04	−0.13	ND	0.59	ND	−0.10	ND	2.71	1.26	0.71	ND	0.02	ND	2.83	1.25	−0.69	ND	2.12	1.59	2.81	2.07
LOC_Os12g17010 PROLM29	−3.22	−2.84	−1.22	−1.39	2.37	9.23	−2.99	−3.25	−2.57	−2.62	2.00	1.46	5.59	12.07	0.23	ND	0.65	ND	3.59	10.62	−1.77	−1.86	−1.35	−1.24	−5.36	−12.48	−4.94	−11.85	0.42	ND
LOC_Os12g16880 PROLM27	−2.54	−2.39	−0.32	ND	2.08	5.95	−2.35	−1.98	0.06	ND	2.22	1.62	4.62	8.34	0.19	ND	2.60	2.09	2.40	6.72	−2.03	−1.21	0.38	ND	−4.43	−7.93	−2.02	−6.24	2.41	1.68
LOC_Os02g16830 Glu	−1.03	−0.55	0.00	ND	1.81	2.39	−0.51	ND	−0.11	0.48	1.03	0.94	2.84	2.94	0.52	0.41	0.92	1.03	1.81	1.99	−0.51	−0.53	−0.11	ND	−2.32	−2.52	−1.92	−1.90	0.40	0.62
LOC_Os02g14600 Glu	−0.54	−0.67	1.22	1.05	0.88	0.43	0.62	ND	0.35	ND	1.76	1.72	1.42	1.10	1.17	0.83	0.90	0.55	−0.34	−0.62	−0.59	−0.89	−0.86	−1.18	−0.25	ND	−0.52	−0.56	−0.27	ND
LOC_Os02g14720 *GW2*	0.05	ND	0.65	0.70	0.82	0.75	0.28	ND	0.29	ND	0.59	0.85	0.77	0.90	0.23	ND	0.24	ND	0.17	ND	−0.36	−0.63	−0.36	−0.43	−0.54	−0.69	−0.53	−0.49	0.01	ND
LOC_Os04g33740 *GIF1*	−0.12	ND	0.34	ND	1.39	1.14	−0.95	−0.66	−0.13	ND	0.46	0.56	1.50	1.41	−0.83	ND	−0.02	ND	1.05	0.85	−1.29	−0.95	−0.47	ND	−2.34	−1.81	−1.52	−1.09	0.82	0.72
LOC_Os07g39480 *WRKY78*	−0.26	ND	−0.06	ND	−0.08	ND	−0.60	−0.46	−1.95	−0.98	0.21	ND	0.19	ND	−0.34	ND	−1.69	−0.93	−0.02	ND	−0.55	−0.65	−1.90	−1.17	−0.53	ND	−1.88	−0.74	−1.35	−0.52
LOC_Os07g08420 *RISBZ1*	0.31	ND	0.56	0.68	0.36	0.46	−0.06	ND	1.10	1.06	0.25	0.57	0.05	ND	−0.37	ND	0.79	0.94	−0.20	ND	−0.62	−0.80	0.54	ND	−0.42	−0.58	0.74	0.60	1.16	1.18

FCs are expressed as log_2_FC, where FC = expression of the gene in the second cv indicated with respect to the first one; ND = not determined

## Discussion

Recent research studies highlighted that each rice grain qualitative trait is governed by numerous QTLs located on different chromosomes [12, 13, 61–63], suggesting that the determination of grain quality is the result of a coordinated regulation of multiple factors. The same conclusion was reached by several large scale comparative studies, based on transcriptomic or proteomic analyses, which lead to the identification of biological processes and genetic determinants affecting rice grain properties. A recent deep RNA sequencing study conducted on two *japonica* rice varieties on six days old developing seeds revealed that milling yield and eating quality are the outcome of the coordination of the biosynthesis of starch, EAs and SSPs [64]. Moreover, a comparative transcriptomic analysis, performed on rice grains, at 15 DAF, from two *japonica* NILs, differing for chalkiness, and a proteomic study carried out to identify differential proteins between the bottom chalky part and the upper translucent part of mature grains of a notched-belly rice mutant, underlined that chalkiness is primarily influenced by genes involved in carbohydrate metabolism, cell wall biosynthesis, redox homeostasis and protein synthesis, assembly and degradation [2, 65].

To identify the genetic factors responsible for the differences in kernels traits among Italian rice cvs, a transcriptomic comparison of developing grains at 14 DAF was here conducted on six among the most renowned Italian rice varieties, CAR, VN, ARB, VOL, BAL and GV. Pair-wise comparisons associated with co-expression analyses of DEGs defined a transcriptional fingerprinting of each cv with the detection of clusters of candidate *loci* responsible for some peculiarities characterizing the six varieties. Enriched GO terms investigations and literature searches classified the *loci* belonging to each cluster as being involved in specific pathways previously demonstrated to affect rice grain quality. Moreover, a deep analysis of all the DEGs in the pair-wise comparisons led to the identification of additional candidates implicated in the determination of starch composition, SSPs and allergens amounts, nutraceutical compounds biosynthesis, grain-size and GT in the six cvs. In the present work a more extended phenotypic characterization was performed on a set of rice genotypes that present a wider genetic variability than described by previous studies. In fact, the studied genotypes show relevant differences in a number of traits important to quality: grain size, AAC, protein content, chalkiness and GT. These differences are reflected in the diverse culinary applications these cv are used for: some (CAR, VN and ARB) are renowned for the preparation of Italian risotto specialties, VOL is used for risotto and timbale, BAL is used for soups and cakes, whereas GV is an old cv used for breeding because of its resistance to diseases.

### Regulation of genes involved in grain chalkiness

According to previous results, 15 DAF is considered a critical time-point for grain filling and for chalkiness determination [2]. Although with contrasting results, Liu et al. (2010) [2] and Lin et al. (2014) [65] stated that a pivotal role in chalkiness determination is attributed to starch and carbohydrate metabolism and in particular to the cell wall biosynthetic processes. In our experiment, DEGs involved in such pathways were mainly up-regulated in CAR: genes encoding for cellulose, β-glucan, SSs and others positively affecting cell wall biosynthesis were detected for clusters 2, 7 and 8. Besides to cell wall biosynthetic enzymes or regulators, cellulose catabolic enzymes, like glucosidases, were also present in clusters 7 and 8 according to the fact that cell walls are subjected to a dynamic remodelling during grain development [66]. Genes encoding for proteins regulating the biosynthesis and the integrity of cell wall were over-expressed also in ARB (sub-cluster 5.1), VOL (cluster 4) and VN (cluster 3). This up-regulation might be responsible for the large chalkiness identified for these four cvs and the highest number of *loci* belonging to this class over-expressed in CAR could influenced the appearance of grain as this cv differentiated form the others for showing not only a large lateral chalkiness but also a central one. VN grain appearance could be also associated to the low transcription levels of the positive regulator of grain dimensions, the cell-wall invertase GIF1 [44], as it was demonstrated that this gene is implicated in chalkiness determination [67]. Differentially from the other genotypes, VOL showed higher transcription of α and β-amylases which action on starch might influence grain appearance [68]. Moreover, with respect to the other clusters, the VOL-specific over-expressed cluster 4 contained the highest number of *loci* implicated in oxidation-reduction processes, like ROS scavenging. Genes of such type were hypothesized playing central roles in chalkiness determination in rice [2]. The co-expression analysis did not reveal a specific over-expression of chalkiness-related *loci* for BAL, suggesting that the small chalkiness detected for this cv is likely associated to the low level of expression of such *loci* (Fig. 3 and Additional file 4, sheets 2 and 3).

### Transcriptional modulation of protein content-related genes

Even protein metabolic processes such as synthesis, folding and degradation assume pivotal functions in grain quality determination. For example, down-regulation of chaperons as BiPs and PDILs, involved in SSPs accumulation, were demonstrated to play essential roles in the occurrence of a floury endosperm in rice chalky grains [65, 69, 70]. Several *loci* over-expressed in CAR belonging to cluster 2 encoded for chaperons and in particular one was the *BiP* gene. A slightly increasing of the levels of the BiP1 protein, encoded by this gene, in rice seeds were accompanied by higher amounts of SSPs, including Glus, in endosperm [69] and, in agreement with these previous observations, the slight up-regulation (about two times) of *BiP* correlates with the highest expression of Glus encoding genes observed for CAR that was detected when analyzing all the DEGs encoding for SSPs identified in the pair-wise comparisons. Conversely, the lowest levels of SSPs in the small and round seed variety BAL could be related to a down-regulation of Glus encoding genes, as Glus represent the 80 % of total SSPs in rice kernels. PROLMs influence on physiochemical properties of rice seeds is not clear, however, experiments carried out for three rice cvs showed that when PROLMs were added to rice flour there was a reduction in adhesiveness of starch gel [71]. Among our varieties, VOL and GV, having both 6.1 % of total proteins in seeds, represented the genotypes showing the highest expression of PROLMs encoding genes. VOL showed the highest expression also for key genes promoting PROLMs accumulation and transcription and encoding for the RBP-B protein [72] and the RISBZ1 bZIP TF [55], respectively. Several *loci* implicated in protein synthesis and metabolism were also present in VOL up-regulated cluster 4, like a glutamine synthetase, a key enzyme influencing total protein amount in seeds [73]. Milling head yield is strictly influenced by protein content [3] and chalkiness [2], however, despite the fact that GV and VOL had the same percentage of grain proteins and the same appearance, GV showed a lower milling head yield with respect to VOL. Considering that milling head yield and grain chalkiness are regulated by the coordination of multiple pathways, other factors could contribute to the differences in milling head yield identified for the two cvs. Despite the fact that VN and ARB displayed the highest total amount of proteins in seeds, they did not show a peculiar over-expression for genes encoding for Glus, excluding LOC_Os02g25860 for ARB, as mentioned in the Results section, or PROLMs. It is likely that the accumulation of SSPs in these cvs could occur at different developmental stages than 14 DAF or that VN and ARB kernels are enriched of other classes of proteins. According to this last hypothesis VN showed up-regulation of proteins carrying LTPL domains, identified in plant lipid transfer proteins, trypsin–α-amylase inhibitors and, more interestingly, in SSPs [57].

### Regulation of starch-related genes

Cooking properties are primarily influenced by starch composition and specifically by the AAC, as low AAC grains become glossy and tender after cooking. AAC is primarily controlled by the gene *Wx* (LOC_Os06g04200) [3, 16]. This gene was more expressed (from 3 to 5 times) in the varieties showing the highest AAC values (CAR, GV and VN) with respect to the others (see Additional file 5), according to the observation that the amount of amylose in the endosperm is directly proportional to the *Wx* transcription level [18]. However, the *Wx* expression in the six genotypes explained only the 8.82 % of phenotypic variance (PC2) in PCA analyses performed considering all the genes affecting starch content and composition identified among the DEGs in the pair-wise comparisons. Nonetheless, our previous analysis underlined that a consistent percentage of variance in AAC (77.5 %) of 125 *japonica* rice accessions was ascribable to the presence of a G/T SNP in the donor splicing site of the leader intron of *Wx*, responsible of a post-transcriptional regulation [16]. Indeed, this SNP pinpoints the two main *Wx* alleles characterized: *Wx*^*a*^ (haplotype G), present in high AAC varieties, and *Wx*^*b*^ (haplotype T), identified in low AAC cvs [19, 20, 74–77]. The presence of the G to T mutation in the *Wx*^*b*^ allele results in the retention of the first intron and the reduction of the amount of functional enzymes in the endosperm determining low AAC phenotypes [78]. Among the six cvs, *Wx*^*b*^ was detected for the ones showing the lowest AAC values (VOL, BAL and ARB) as they showed the retention of the intron due to the presence of the SNP, while the other genotypes, with high AAC, carried *Wx*^*a*^ as a complete splicing was exhibited. By this view, according to our previous analyses [16], the variation in AAC detected among our genotypes could be associated to the presence of the two alleles at the *Wx locus*, which not only show a differential splicing but also different expression rates, thus leading to variation in the amount of GBSSI functional enzymes in the endosperm.

The only covalent modification of starch is phosphorylation which alters its physical properties and is in part responsible of a sticky grain [79]. The reaction is catalyzed by the α-Glucan Water Dikinase (GWD) enzyme which transfers the β-phosphate of ATP to glycolyl residues of starch [15, 80]. In our case, the *GWD* gene (LOC_Os06g30310) showed lower expression for the two main Italian risotto varieties CAR and VN, making possible that lower expression of this gene affects the cooking properties of the grains of these two cvs.

Another factor affecting rice eating and cooking properties is the GT which depends on the degree of polymerization of amylopectin chains. The main determinant of GT is the SSIIa enzyme and, in particular, two functional SNPs at the level of the SSIIa encoding *locus* were characterized as responsible for low GT: one at +4,198 bp from the start codon, known to inactivate the SSIIa enzyme [22], and the other, at position +4,330, typical of low GT rice genotypes [81]. The low GT old cv GV differentiated from the other varieties for the presence of the first SNP, which is quite rare [4]. The presence of the second SNP was identified in CAR, VOL and BAL and could be associated to the low alkali values detected for these varieties. Nonetheless, this mutation was also observed in the intermediate GT ARB and VN. However, it is important to consider that the control of intermediate GT in rice is still mostly unknown and it has been highlighted that other unknown enzymes play important roles in determining amylopectin chains polymerization [82].

### Grain size-related genes

Grain quality is strictly associated with grain size, which is influenced by a complicate and still poorly understood cooperation of many genes, some of which identified and annotated in rice genome databases [44]. Considering all the DEGs in the pair-wise comparisons we could identify candidates that might play pivotal roles in grain size determination for our cvs. The longest size of naked seeds were recorded for CAR and it could be related to the up-regulation of the positive regulators of grain length *D61*, *WRKY78* and *SRS1* [43, 44, 59] (see Additional file 5). Moreover the slimmer long A naked grains of CAR and GV might be associated with the over-expression of the negative regulator of grain width *GW2* [58] (see Additional file 5). The down-regulation in VN of the positive regulators of seed dimensions *GIF1* [44] and the large subunit of AGPase, positively linked to seed weight [53] (see Additional file 5), could contribute to the VN medium grain. As indicated for the *Wx* and *SSIIa loci*, the power of RNA-Seq is not limited to the definition of DEGs but it is also useful in the detection of putative functional SNPs. Exploiting this possibility, we observed that BAL carries a single SNP located in exon four of the *SRS5* gene and demonstrated to be responsible of a small and round seed [60], suggesting that this mutation is causal of BAL round grain. Moreover, this cv showed the highest milling head yield, which could be associated to grain size, as small and round seeds are more resistant to milling with respect to long grains [83–85], and to the low level of chalkiness, as chalky grains are more prone to breakage during milling in comparison to vitreous ones [65].

### Modulation of nutraceutical- and allergens-related genes

Rice nutritional quality is also dependent on the content of lipids, which affects storage stability [4], EAs, VTE components, γ-oryzanol and other nutraceutical compounds. Up-regulation of genes involved in lipid metabolism was detected mainly for VOL and, interestingly, in VOL up-regulated cluster 4 we identified a *locus* encoding for a terpene synthase, a key enzyme for the biosynthesis of γ-oryzanol. However, a linear correlation between transcriptomic and phenotypic analyses was not revealed considering all the DEGs in the pair-wise comparisons encoding for enzymes involved in tocochromanols and γ-oryzanol biosynthetic pathways. This might be due to an earlier regulation of such genes with respect to 14 DAF [86]. In addition, very little is known about the control of the accumulation of these compounds in seeds and it is also possible that uncharacterized regulators could play essential roles. Clusters analyses revealed that VN showed the highest up-regulation of genes encoding for nutritional proteins, or affecting nutraceutical compounds biosynthesis, followed by CAR, VOL and ARB. Moreover, our results highlighted that each variety showed a peculiar transcriptional regulation of a set of genes related to specific nutraceutical compounds that included glucans for CAR, thiamins for ARB, alkaloids, flavonoids, terpenoids and glicans for VN, THIONs for BAL and flavonols and terpenoids for VOL. A cv-specific transcriptional profile was observed even for genes involved in EAs biosynthesis, since ARB showed up-regulation of *loci* for Trp biosynthesis with respect to the other genotypes, while GV had higher transcription of genes for Asp and His biosynthesis. Conversely, VOL showed the highest up- and down-regulations of DEGs involved in EAs degradation and biosynthesis, respectively. More detailed phenotypic analyses will be necessary to assess whether the different modulation of EAs associated genes among our varieties results in different contents of the corresponding EAs.

Even for *loci* encoding for known allergens or classes of allergenic proteins, the co-regulation analysis and the detailed searches of all the DEGs in the pair-wise comparisons revealed a specific transcriptional regulation for each variety. The low seed protein content cv BAL represents the genotype carrying the lowest expression for allergens. Conversely, higher transcription for cupin domain containing proteins and LTPL encoding genes was detected for VN which showed the highest expression also for genes encoding two known allergens: OsSub31 and ppg4. Some LTPL up-regulation was also detected for CAR and VOL displaying higher transcription also for albumins. Moreover, VOL showed over-expression for one of the main rice allergens, RAL6 which accumulates at 15–20 DAF [45].

### Integrated analyses and data validation

The co-regulation analysis identified clusters characterized by many genes linked with multiple connections underlining tight relationships. Considering this aspect, genes belonging to the same cluster might cooperate together in defining the peculiar characteristics of a certain variety. Indeed, analyzing the closest connections inside a cluster, it is possible to hypothesize putative roles for specific genes. As an example, in cluster 2, through the homeobox LOC_Os01g70810, the cellulose synthase CSLC9 (LOC_Os03g56060) was tightly associated with the MYB TF LOC_Os05g03550 and with the C-x8-C-x5-C-x3-H zinc finger LOC_Os03g02160. MYB TFs are known to regulate cell wall biosynthesis in many species like poplar [87], *Pinus* [88], maize, *A. thaliana* [89–91] and rice, for which OsMYB58/63 were recently reported to activate the expression of the cellulose synthase OsCesA7 [92]. Moreover, in rice and maize, MYB46 was demonstrated to activate the cell wall biosynthetic pathway [46] and its homolog in *Arabidopsis* triggers the expression of cellulose synthases directly [93] and indirectly throughout the activation of a C-x8-C-x5-C-x3-H zinc finger encoding gene [94, 95]. Even if LOC_Os05g03550 does not correspond to the MYBs 46, 58 or 63, the tight co-regulation with CSLC9 and LOC_Os03g02160 could suggest a putative role in cell wall biosynthesis regulation for this *locus*. The same could be hypothesized also for the MYB LOC_Os03g22560, which is closely related to the C3HC4 type zinc finger LOC_Os10g39770 and the trehalose synthase LOC_Os01g53000. In addition, within cluster 2, cell wall biosynthetic enzymes and a FERONIA receptor kinases, belonging to a class of proteins regulating cell wall biosynthesis [42] as reported above, were tightly transcriptionally co-regulated with signal factors like STRUBBELIG–RECEPTOR, MAPK, CrRLK1L, GATA zinc finger and mps, involved in a number of processes like hormone signalling, biotic and abiotic stress response, homeostasis, development and protein-protein interactions [96–98]. This association might reveal hypothetical involvements of these regulatory genes also in the regulation of cell wall biosynthesis.

RNA-Seq data were confirmed by qRT-PCR analyses and the same patterns of expression of selected *loci* was observed as those detected in the pair-wise comparisons. Duan and Sun (2005) [31] performed a time course analysis of starch biosynthetic genes in two *indica* varieties highlighting that transcription of most of them initiate at 3 DAF, peaked at 10 DAF and started to decline at 15 DAF. Exceptions to this trend were represented by *Wx* which showed two picks, at 6 and 15 DAF, and by the small subunit of AGPase, which peaks at 6 DAF and then declined slowly towards maturation. In our experiment, *Wx* was more expressed at both 10 and 14 DAF with respect to 6 DAF, with higher mRNA levels at 14 DAF for all the six cvs. The same behaviour was observed for the other main determinants of starch content in seeds, the two subunits of AGPase. Our results are however in agreement with the higher expression of *Wx* and *AGPases* observed at 15 DAF in the *japonica* cvs analysed by Venu and co-workers (2011) [64]. In addition, qRT-PCR results highlighted a different time-dependent regulation for the α-Glucan Water Dikinase *GWD* gene (LOC_Os06g30310) in the two risotto cvs CAR and VN, with respect to the other genotypes. Indeed, the two varieties showed a down-regulation at 14 DAF while, for the other cvs, an increased *GWD* expression at 14 DAF was observed. The regulation of starch phosphorylation, through the modulation of *GWD* expression, can provide to CAR and VN peculiar cooking properties to grains. Differential time-course expression was found for the SSPs encoding genes considered, according to previous analyses revealing that the transcription of SSPs-encoding genes is *locus*-specific, with some peaking at 10 DAF, and some at 15 DAF, although maintaining a significant expression activity at late developmental stages [31]. Moreover, the different transcription patterns for SSPs observed among the six varieties suggest a peculiar regulation of specific SSPs *loci* in each cv. More detailed phenotypic assessments of the specific classes of SSPs present in the grains of the six accessions are needed to provide a better understanding of the effect of the transcriptional regulation.

## Conclusions

This work represents the first analysis leading to a transcriptional fingerprinting of the most renowned Italian rice varieties, providing a gene-expression based picture for most of the grain quality differences observed among the cvs analyzed. The results revealed that each variety is characterized by a peculiar transcriptional regulation of specific sets of genes involved in pathways affecting grain appearance and quality. With respect to the other genotypes, the chalky, long A, high AAC caryopsis of CAR was characterized by a high expression of classes of *loci* involved in chalkiness determination, like carbohydrate metabolism and cell wall biosynthesis, and of genes encoding for Glus, GBSSI, GWD, some positive regulators of grain length and the negative regulator of seed width GW2; VOL, showing a chalky, long A, low AAC grain, was characterized by a high transcription of chalkiness-related *loci* implicated in cell wall biosynthesis and oxidation-reduction processes and of PROLMs encoding genes. Moreover, this cv displayed low expression for *Wx* and was the genotype carrying the highest modulation of genes implicated in EA biosynthesis and degradation; the vitreous, small and round and low AAC seed of BAL showed low levels of mRNAs of *Wx*, genes implicated in chalkiness determination and *loci* encoding for allergens and high transcription of a number of defense response genes; for the chalky, medium, high AAC VN grain, an high level of expression of *Wx*, *GWD* and the majority of *loci* encoding for nutraceutical compounds and allergens was detected. Furthermore, VN showed down-regulation of positive regulators of grain size, with respect to most of the other genotypes; GV, carrying a chalky, long A and high AAC grain, displayed high transcription of some *loci* implicated in defense response, the *Wx* gene, *loci* encoding for PROLMs or involved in protein metabolism, a class of genes affecting chalkiness; the chalky, long A, low AAC grain of ARB was characterized by high expression of *loci* implicated in response to abiotic stress, protein metabolism and redox homeostasis. Despite the fact that a complete knowledge of all the factors determining grain quality and how they interact is still missing, a list of candidates affecting the specific grain properties of each variety with respect to the others on the base of differential expression was obtained. Moreover, besides the differential transcriptional regulation among the six cvs, putative functional SNPs associated to specific phenotypic traits were detected. The identification of candidate genes responsible for qualitative traits of the main Italian rice varieties offers a starting point for further works aimed to the characterization of *loci* and molecular markers associated with quality to be used in breeding programs.

## Methods

### Plant materials and RNA sequencing

The six traditional Italian *japonica* varieties Carnaroli (CAR, long A, released in 1945), Arborio (ARB, long A, released in 1946), Vialone Nano (VN, medium, released in 1937), Gigante Vercelli (GV, long A, released in 1946), Balilla (BAL, small and round, released in 1924) and Volano (VOL, long A, released in 1968) were grown in greenhouse under flooding conditions at an average of 26 °C during the day and at about 22 °C during the night. Developing seeds were sampled at 14 DAF, an intermediate time for the accumulation of nutritional compounds in the grains. Three biological replicates, each represented by five panicles obtained from different plants (approximately 200 developing grains per replicate), were performed for each genotype. Total RNA was extracted following the protocol described by Li and Trick (2005) [99]. RNA-Seq libraries were prepared by the TruSeq RNA sample preparation kit (Illumina), according to manufacturers’ instructions. Libraries were quantified through qRT-PCR, as recommended by the protocol, and single-end sequenced for 70 bases on an Illumina Genome Analyzer (GAIIx).

### Bioinformatic and statistical methods

Raw fastQ files were checked for low-quality reads and contaminants via fastQC application. Reads containing contaminant primer/adapters and long stretches of poor quality bases were removed. Low quality reads (quality < = 10 phred score) and contaminants were trimmed out with the Cutadapt software [100]. Contaminant-free, filtered reads were mapped with Bowtie/Tophat version 1.4.1 [101] to the rice genome (*O. sativa* Nipponbare MSU 6.16 release). Based on rice small intron size [102], minimum and maximum intron length of 30 and 50,000, respectively, were set. Read counts were collected from the BAM alignment files with HTSeq version 0.5.3 [103] in the single-end and ‘union’ mode using the *O. sativa* MSU 6.16 gtf file as obtained from the Ensembl Plant Repository.

#### DEG calling

The DESeq package [104] version 1.6.0 was used under R release 2.14 to identify differentially expressed genes (DEGs). Raw read counts as obtained by the HTSeq application were normalized to RPKM (Reads per Kilobase per Million) and genes above the 0.1 RPKM threshold were considered expressed. Moreover, as for DESeq default setting, a combination of fitted versus per gene dispersion values was implemented. The FDR threshold for DEG calling was set to 0.05.

#### GO term enrichment analyses

The goseq bioconductor package was employed since it was developed to account for RNA length bias typical of RNA-Seq approaches [105, 106]. As rice database are not yet covered by goseq, a Bioconductor package, gene lengths were retrieved with BiomaRt queries (*Oryza sativa* MSU 6.16) out of rice Nipponbare cDNA and median length for each rice *locus* were obtained by parsing cDNA length data with R custom scripts. Gene ontology terms for CC (cellular components), MF (molecular functions) and BP (biological processes) were similarly retrieved with BiomaRT queries. An FDR cutoff of 0.05 was used for GO enrichments.

#### Co-expression analyses

A matrix of 10,200 rows (genes called as DEG in at least one of the contrasts and, additionally exhibiting a fold change of at least 2) and as columns all 18 samples (three biological replicates for each 6 genotypes) was generated by subsampling the whole DESeq-normalized matrix of expression data (countSet). For such matrix, signed network adjacency was calculated implementing the Adjacency function as available in R WGCNA package (version 1.46) [107]. Correlation threshold was set to 0.9. To obtain edges and nodes, a graphNEL-type graph was subsequently generated from the adjacency matrix and sent via the Rcytoscape Bioconductor package [108] to Cytoscape application version 2.8.1 [109] for cluster visualization and analysis. Functions from Rcytoscape package were used in order to annotate, select, extend and communicate back relevant data to R for batch cluster analyses with custom R scripts. Biological Process enriched GOs for genes in cluster were calculated with the hypergeometric test as implemented in Bioconductor GOstats package [110] using a *p*-value cutoff of 0.05.

#### Isoform analysis

The identification of putative alternative splicing forms was conducted with Bioconductor DEXSeq package (v1.11.16) [111]. As a prerequisite, *O. sativa* Nipponbare MSU 6.16 gtf file was modified by deseq_prepare_annotation.py and dexseq_count.py as detailed in DEXSeq package documentation. Visualization of alternative splicing was made by plotDEXSeq function as available in DEXSeq package.

#### Principal component analysis (PCA)

PCA was performed on the covariance matrix for the differentially expressed genes belonging to the different groups across all the pair-wise comparisons using a custom R script based on the “bpca” R package [112].

### Phenotypic evaluations

For all the phenotypic parameters, three biological replicates for each genotype were assayed using plants grown in greenhouse on paddy soil, under the same environmental conditions as those used for RNA-Seq. Plants were organized in a randomized block experiment in which each replicate was represented by three lines with 20 seeds each sowed at a distance of about 15 cm. AAC was quantified according to the method of Williams et al. (1958) [113], with modifications by Inatsu (1988) [114], by means of a FOSS FIAstar 5000 auto-analyzer, as described by Biselli et al. (2014) [16]. Protein content was determined by the Kjeldahl digestion method [115] which involves the conversion of organic nitrogen into ammonium by boiling with sulphuric acid and distilling with alkali to liberate ammonia for determination by titration. For this analysis, we used 1 g of sample and 1 g of a catalyzer containing Selenium. Seed biometrics was measured on 100 seeds per replicate. Images were acquired with a digital scanner (400 dpi resolution) on both hulled and naked caryopsis and then analyzed using the WinSEEDLE software (Regents Instruments Inc.). Tocochromanols and γ-oryzanol contents were established on 100 mg of bran following the HPLC protocol of Chen and Bergman (2005) [116]. The calibration was performed with a standard mix of pure standards of each tochochromanol and γ-oryzanol in the same concentration. Three calibration points were obtained from three different dilutions of the standard mix: 1/1, ½ and ¼. Milling head yield data were taken by the releases from Ente Nazionale Risi (http://www.enterisi.it). GT was detected by the alkali test described by Bhattacharya and Sowbhagya (1972) [117], considering a scale from 1 to 7: 1–2 high (74.5-80 °C), 3 high intermediate (74–74.5 °C), 4–5 intermediate (70-74 °C), and 6–7 low (<70 °C). Chalkiness was measured for 100 white grains for each of the three biological replicates of each cv with the following scale: 0 = none, 1 = small (<10 %), 5 = medium (10-20 %) and 9 = large (>20 %) [4].

### Quantitative RT-PCR analyses

Two-step quantitative RT-PCRs (qRT-PCRs) were carried out using the same RNAs utilized for the RNA-Seq experiment and additional RNAs extracted by using the same protocol described above from caryopsis at 6 and 10 DAF. Three biological replicates, with the corresponding three technical replicates, were performed for each time-point. cDNAs were synthesized with the Super Script II enzyme (Invitrogen) following manufacturers’ instructions and quantified through the Qubit Fluorometer (Invitrogen). The endogenous control was represented by rice Ubiquitin conjugate enzyme E2 (*UBC*; AK059694). To identify the best housekeeping gene for qRT-PCR analyses, all the genes listed by Jain and co-workers (2006) [118] were tested for our samples using the same primer pairs indicated in the paper. A good standard curve was obtained for five genes: ACT11, UBC, GAPDH, UBQ5 and 18S rRNA (data not shown). For these genes, qRT-PCRs were conducted for all the 18 cDNAs, performing three technical replicates. The lowest cycle threshold (Ct) variation among the different samples was obtained for UBC which was chosen as the housekeeping standard (see Additional file 8). With the exception of *UBC*, for which the same primers designed by Jain and co-workers (2006) [118] were utilized, primers were designed by the Primer3 software (http://simgene.com/Primer3) (see Additional file 9). Blast searches were performed to verify the specificity of each primer for the corresponding gene. As control, the primer pairs were used to perform PCRs on genomic DNA of the six cvs extracted from leaves following the CTAB method [119]. PCRs were carried out in 20 μl of reaction using the KAPA SYBR FAST ABI Prism qPCR Kit (ResnovA) according to manufacturers’ instructions. Relative gene expression was calculated with the 2^-ΔΔCt^ method on the averages of three technical replicates for the three biological replicates [120], performing the same pair-wise comparisons as for the RNA-Seq experiment for each time-point. To analyze the time-course expression of each gene in rice caryopsis, comparisons of transcription levels of the genes at 10 and 14 DAF with respect to 6 DAF were performed for each genotype.

### Ethical standards

This article does not contain any studies with human participants or animals performed by any of the authors.

## Additional files

Additional file 1:
**Number of reads obtained for each replicate of the six cvs.** (DOCX 12 kb) Additional file 2:
**Pearson correlation coefficients for all the three biological replicates corresponding to each cv.** (PDF 37 kb) Additional file 3:
**Heat map representing samples clustering.** (PDF 262 kb) Additional file 4:
**Co-expression analysis results.** (XLSX 1249 kb) Additional file 5:
**RNA-Seq results for DEGs belonging to the main classes of genes affecting rice grain quality.** (XLSX 427 kb) Additional file 6:
**Principal Component Analysis (PCA) based on the expression of starch-related DEGs.** (PDF 39 kb) Additional file 7:
**qRT-PCRs log**_2_ **FCs for the pair-wise comparisons at 6, 10 and 14 DAF.** (DOCX 28 kb) Additional file 8:
**qRT-PCR results for the housekeeping genes, showing the best standard curves, selected among the genes indicated by Jain et al. (2006)** **[** 118 **].** (DOCX 14 kb) Additional file 9:
**Sequence of the primer pairs used for each** ***locus*** **analysed by qRT-PCRs.** (DOCX 12 kb)

## Abbreviations

AAC: Apparent amylose content
AGPase: ADP-Glucose pyrophosphorylase
ARB: Arborio
BAL: Balilla
BE: starch Branching Enzyme
BR: Brassionosteroid
CAR: Carnaroli
CHIT: Chitinase
cv: cultivar
cvs: cultivars
DAF: Days After Flowering
DBE: starch Debranching Enzyme
DEG: Differentially expressed gene
DW: Dry weight
EA: Essential amino acid
FC: Fold Change
FDR: False Discovery Rate
GBSSI: Granule bound starch synthase I
Glu: Glutelin
GO: Gene onthology
GT: Gelatinization temperature
GV: Gigante Vercelli
GWD: α-Glucan Water Dikinase
IGV: Integrative Genome Viewer
LTP: Lipid Transfer Protein
NB-LRR: Nucleotide Binding-Leucine Rich Repeat
PB: Protein Body
PROLM: Prolamin
QTL: Quantitative Trait *Locus*
RPKM: Reads per Kilobase per Million
SNP: Single Nucleotide Polymorphism
SS: Starch Synthase
SSP: Seed Storage Protein
TF: Transcription Factor
THION: Thionin
VN: Vialone Nano
VOL: Volano
*VTE* genes: Vitamin E genes
*Wx*: *Waxy*
