# Supplementary material for: Deep sequencing transcriptional fingerprinting of rice kernels for dissecting grain quality traits
Source: BMC Genomics. 2015 Dec 21;16:1091. doi: 10.1186/s12864-015-2321-7 (PMC4687084; doi:10.1186/s12864-015-2321-7)
Supplement: Additional file 1: — Number of reads obtained for each replicate of the six cvs. (DOCX 12 kb) [file 12864_2015_2321_MOESM1_ESM.docx]

**Additional file 1:** Number of reads obtained for each replicate of the six cvs..

Filtering was performed by using HTSeq and reads were aligned to Nipponbare Reference Genome by Bowtie/tophat. Amounts of unique reads and reads mapping at only one *locus* are also reported.

| **Sample** | **Total filtered reads** | **total alignments** | **unique reads** | **reads with only one mapping** |
| --- | --- | --- | --- | --- |
| ARB 1 | 17245655 | 19791874 | 16417038 | 14306486 |
| ARB 2 | 17097433 | 18867023 | 16259065 | 14346341 |
| ARB 3 | 16466565 | 18545818 | 15671282 | 13714580 |
| BAL 1 | 20375092 | 21686268 | 19433127 | 17815521 |
| BAL 2 | 17770323 | 21713407 | 16947534 | 14941717 |
| BAL 3 | 17827368 | 20554729 | 17015161 | 15256656 |
| CAR 1 | 16084775 | 18215217 | 15340776 | 13394861 |
| CAR 2 | 18497480 | 21931213 | 17574419 | 15192381 |
| CAR 3 | 18745984 | 21712427 | 17884519 | 15539936 |
| GV 1 | 19187658 | 21943491 | 18330207 | 15624647 |
| GV 2 | 19392508 | 22535512 | 18547576 | 15542810 |
| GV 3 | 19796567 | 22731017 | 18919371 | 15983047 |
| VN 1 | 30501367 | 34171629 | 29626908 | 26491048 |
| VN 2 | 35722029 | 42527813 | 34693238 | 30433663 |
| VN 3 | 17863813 | 19444603 | 16416986 | 14569500 |
| VOL 1 | 30881988 | 34978339 | 28899373 | 25465668 |
| VOL 2 | 34071882 | 39791063 | 32952142 | 28907249 |
| VOL 3 | 17881725 | 20612722 | 17044076 | 14921233 |

ARB-Arborio, BAL-Balilla, CAR-Carnaroli, GV-Gigante Vercelli, VN-Vialone Nano, VOL-Volano.
