# Supplementary material for: Deep sequencing transcriptional fingerprinting of rice kernels for dissecting grain quality traits
Source: BMC Genomics. 2015 Dec 21;16:1091. doi: 10.1186/s12864-015-2321-7 (PMC4687084; doi:10.1186/s12864-015-2321-7)
Supplement: Additional file 2: — Pearson correlation coefficients for all the three biological replicates corresponding to each cv. (PDF 37 kb) [file 12864_2015_2321_MOESM2_ESM.pdf]

Additional file 2: Pearson correlation coefficients for the three biological replicates corresponding to each cv.

	ARB 1	ARB 2	ARB 3	BAL 1	BAL 2	BAL 3	CAR 1	CAR 2	CAR 3	GV 1	GV 2	GV 3	VN 1	VN 2	VN 3	VOL 1	VOL 2	VOL 3
**ARB 1**	1.0000	0.9999	0.9997	0.9645	0.9799	0.9835	0.9777	0.9777	0.9785	0.9783	0.9797	0.9812	0.9757	0.9747	0.9671	0.9655	0.9744	0.9735
**ARB 2**	0.9999	1.0000	0.9997	0.9660	0.9800	0.9841	0.9789	0.9789	0.9797	0.9785	0.9802	0.9817	0.9759	0.9748	0.9672	0.9662	0.9750	0.9741
**ARB 3**	0.9997	0.9997	1.0000	0.9655	0.9789	0.9835	0.9793	0.9788	0.9798	0.9774	0.9806	0.9819	0.9761	0.9753	0.9665	0.9666	0.9759	0.9750
**BAL 1**	0.9645	0.9660	0.9655	1.0000	0.9591	0.9816	0.9754	0.9765	0.9752	0.9615	0.9712	0.9700	0.9728	0.9724	0.9633	0.9416	0.9549	0.9538
**BAL 2**	0.9799	0.9800	0.9789	0.9591	1.0000	0.9954	0.9610	0.9627	0.9625	0.9739	0.9631	0.9709	0.9699	0.9704	0.9593	0.9849	0.9853	0.9858
**BAL 3**	0.9835	0.9841	0.9835	0.9816	0.9954	1.0000	0.9749	0.9763	0.9758	0.9787	0.9752	0.9800	0.9803	0.9806	0.9695	0.9806	0.9855	0.9855
**CAR 1**	0.9777	0.9789	0.9793	0.9754	0.9610	0.9749	1.0000	0.9997	0.9999	0.9722	0.9790	0.9792	0.9739	0.9725	0.9672	0.9564	0.9675	0.9664
**CAR 2**	0.9777	0.9789	0.9788	0.9765	0.9627	0.9763	0.9997	1.0000	0.9998	0.9744	0.9795	0.9800	0.9752	0.9737	0.9693	0.9570	0.9674	0.9664
**CAR 3**	0.9785	0.9797	0.9798	0.9752	0.9625	0.9758	0.9999	0.9998	1.0000	0.9735	0.9795	0.9799	0.9742	0.9727	0.9677	0.9575	0.9680	0.9670
**GV 1**	0.9783	0.9785	0.9774	0.9615	0.9739	0.9787	0.9722	0.9744	0.9735	1.0000	0.9933	0.9970	0.9875	0.9866	0.9819	0.9623	0.9676	0.9666
**GV 2**	0.9797	0.9802	0.9806	0.9712	0.9631	0.9752	0.9790	0.9795	0.9795	0.9933	1.0000	0.9988	0.9878	0.9875	0.9783	0.9540	0.9641	0.9626
**GV 3**	0.9812	0.9817	0.9819	0.9700	0.9709	0.9800	0.9792	0.9800	0.9799	0.9970	0.9988	1.0000	0.9898	0.9893	0.9812	0.9623	0.9705	0.9693
**VN 1**	0.9757	0.9759	0.9761	0.9728	0.9699	0.9803	0.9739	0.9752	0.9742	0.9875	0.9878	0.9898	1.0000	0.9994	0.9947	0.9626	0.9716	0.9709
**VN 2**	0.9747	0.9748	0.9753	0.9724	0.9704	0.9806	0.9725	0.9737	0.9727	0.9866	0.9875	0.9893	0.9994	1.0000	0.9909	0.9644	0.9731	0.9725
**VN 3**	0.9671	0.9672	0.9665	0.9633	0.9593	0.9695	0.9672	0.9693	0.9677	0.9819	0.9783	0.9812	0.9947	0.9909	1.0000	0.9472	0.9564	0.9556
**VOL 1**	0.9655	0.9662	0.9666	0.9416	0.9849	0.9806	0.9564	0.9570	0.9575	0.9623	0.9540	0.9623	0.9626	0.9644	0.9472	1.0000	0.9974	0.9978
**VOL 2**	0.9744	0.9750	0.9759	0.9549	0.9853	0.9855	0.9675	0.9674	0.9680	0.9676	0.9641	0.9705	0.9716	0.9731	0.9564	0.9974	1.0000	0.9998
**VOL 3**	0.9735	0.9741	0.9750	0.9538	0.9858	0.9855	0.9664	0.9664	0.9670	0.9666	0.9626	0.9693	0.9709	0.9725	0.9556	0.9978	0.9998	1.0000

ARB-Arborio, BAL-Balilla, CAR-Carnaroli, GV-Gigante Vercelli, VN-Vialone Nano, VOL-Volano.
