# Supplementary material for: Deep sequencing transcriptional fingerprinting of rice kernels for dissecting grain quality traits
Source: BMC Genomics. 2015 Dec 21;16:1091. doi: 10.1186/s12864-015-2321-7 (PMC4687084; doi:10.1186/s12864-015-2321-7)
Supplement: Additional file 3: — Heat map representing samples clustering. (PDF 262 kb) [file 12864_2015_2321_MOESM3_ESM.pdf]

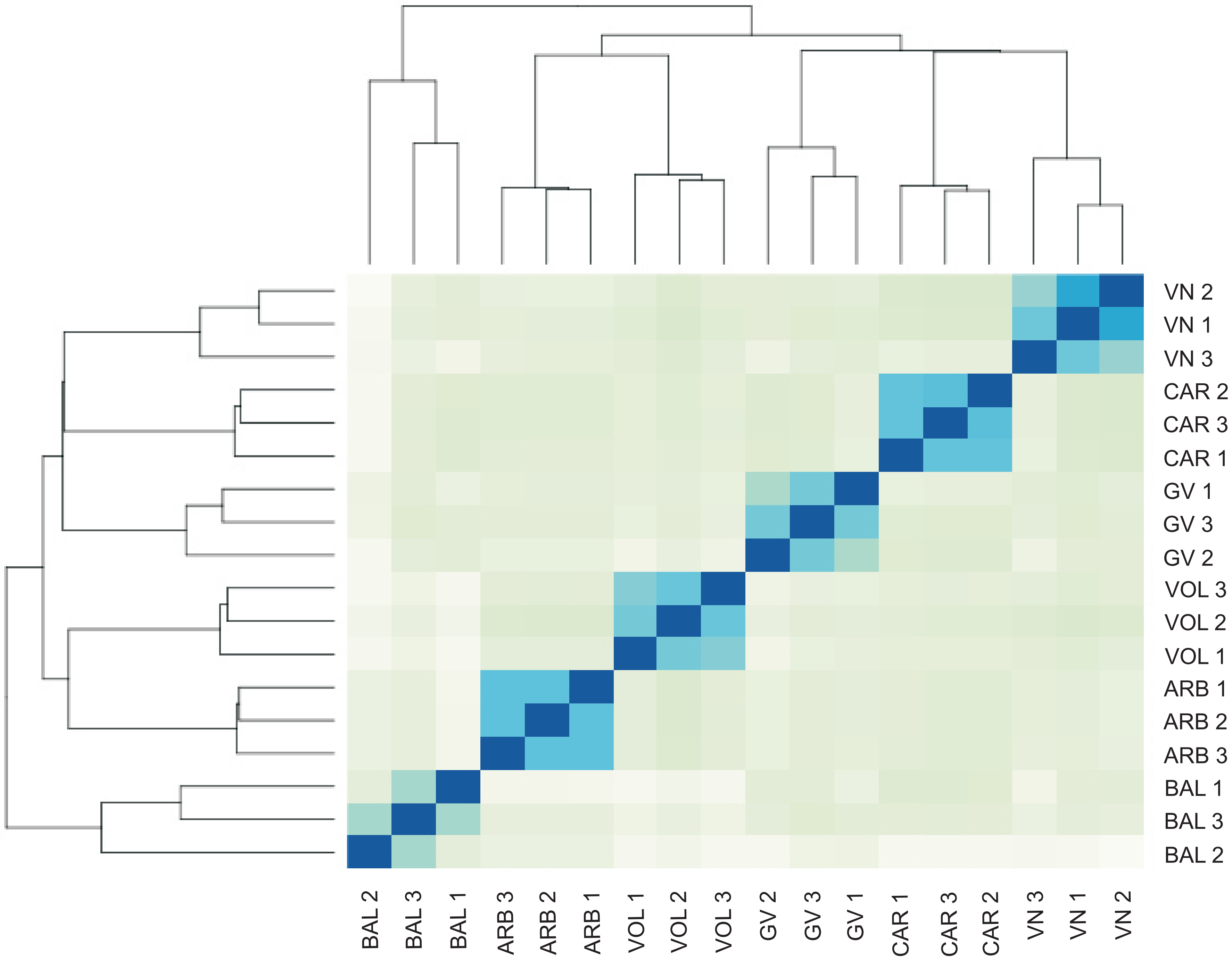

Additional file 3: Heat map representing samples clustering. The Pearson correlation coefficients for the three biological replicates of each variety are schematized. Prior to hierarchical clustering, expression data were transformed with VST (DESeq R package) function. Colour scale indicates the degree of correlation: light blue for higher correlation, dark blue for lower correlation.
