# Supplementary material for: Deep sequencing transcriptional fingerprinting of rice kernels for dissecting grain quality traits
Source: BMC Genomics. 2015 Dec 21;16:1091. doi: 10.1186/s12864-015-2321-7 (PMC4687084; doi:10.1186/s12864-015-2321-7)
Supplement: Additional file 6: — Principal Component Analysis (PCA) based on the expression of starch-related DEGs. (PDF 39 kb) [file 12864_2015_2321_MOESM6_ESM.pdf]

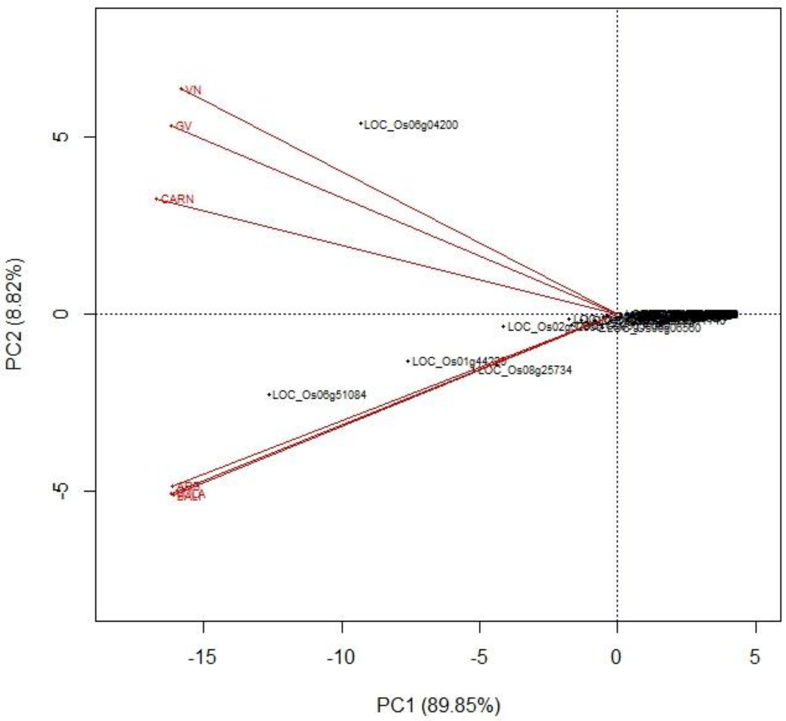

Additional file 6: Principal Component Analysis (PCA) based on the expression of starch-related DEGs. Only DEGs belonging to the GO terms starch metabolism, starch biosynthesis and starch catabolism were considered. The loading (explained variation) of each relevant component for the considered gene set (dependent variable) is shown. Moreover, the expression of each gene is centered (i.e. the origin of the axes is set at 0,0) on the average expression level of the set of genes considered, over all the six genotypes. Genes that showed an overall level of expression different from the average, as well as genes that were differentially expressed among genotypes, were responsible of the variation detected by the PCA, and then appear to deviate from the centre of the plot. The farthest away a gene is positioned from the centre of the plot along a component, the more relevant is its incidence in explaining the variability picked up by that component. Typically, the PC1 picked up differences owed to the presence of some genes that were much more expressed than the others, whereas the PC2 picked up differences in the expression level among the cvs..
