# Supplementary material for: Deep sequencing transcriptional fingerprinting of rice kernels for dissecting grain quality traits
Source: BMC Genomics. 2015 Dec 21;16:1091. doi: 10.1186/s12864-015-2321-7 (PMC4687084; doi:10.1186/s12864-015-2321-7)
Supplement: Additional file 7: — qRT-PCRs log 2 FCs for the pair-wise comparisons at 6, 10 and 14 DAF. (DOCX 28 kb) [file 12864_2015_2321_MOESM7_ESM.docx]

**Additional file 7:** qRT-PCRs log_2_FCs for the pair-wise comparisons at 6, 10 and 14 DAF.

Values were obtained comparing the expression values of the second cv. indicated with respect to the ones of the first cv..

| ***Locus*** | **ARB/BALI** | **ARB/CARN** | **ARB/GV** | **ARB/VN** | **ARB/VOLA** | **BALI/CARN** | **BALI/GV** | **BALI/VN** | **BALI/VOLA** | **CARN/GV** | **CARN/VN** | **CARN/VOLA** | **GV/VN** | **GV/VOLA** | **VN/VOLA** |
| --- | --- | --- | --- | --- | --- | --- | --- | --- | --- | --- | --- | --- | --- | --- | --- |
|  | **6 DAF** |  |  |  |  |  |  |  |  |  |  |  |  |  |  |
| LOC_Os06g04200  *GBSSI* | -0.82 | 0.17 | 0.83 | 1.35 | -0.36 | 0.98 | 1.65 | 2.16 | 0.45 | 0.66 | 1.18 | -0.53 | 0.52 | -1.19 | -1.71 |
| LOC_Os06g30310  *GWD* | -2.66 | -2.05 | -1.46 | -1.05 | 0.03 | 0.61 | -0.85 | -0.44 | -0.41 | -1.46 | -1.05 | -1.02 | 0.41 | 0.44 | 0.03 |
| LOC_Os05g50380  *OsAGPaseL* | 1.57 | 0.75 | 1.17 | 0.21 | 0.85 | 1.16 | 0.87 | -0.15 | 1.32 | -0.11 | -0.52 | 0.85 | -0.87 | 0.57 | 1.58 |
| LOC_Os08g25734  *OsAGPaseS* | 2.24 | 1.14 | 1.28 | -2.09 | 2.75 | -1.10 | 0.18 | -3.19 | -0.44 | 1.28 | -2.09 | 0.66 | -3.38 | -0.63 | 2.75 |
| LOC_Os07g11910  PROLM20 | -0.57 | -0.91 | 1.07 | 0.46 | 1.24 | -0.34 | 0.74 | 0.12 | 1.36 | 1.07 | 0.46 | 1.70 | -0.62 | 0.62 | 1.24 |
| LOC_Os07g10570  PROLM25 | -1.33 | -1.22 | -0.38 | 0.70 | 0.65 | 0.11 | -0.27 | 0.81 | 1.46 | -0.38 | 0.70 | 1.35 | 1.08 | 1.73 | 0.65 |
| LOC_Os12g17010  PROLM29 | -1.30 | -0.31 | -0.76 | -0.30 | 1.06 | 0.99 | 0.23 | 0.69 | 1.75 | -0.76 | -0.30 | 0.76 | 0.46 | 1.51 | 1.06 |
| LOC_Os12g16880  PROLM27 | -1.73 | -0.73 | 0.38 | 0.25 | 0.74 | 0.99 | 1.37 | 1.24 | 1.98 | 0.38 | 0.25 | 0.98 | -0.13 | 0.60 | 0.74 |
| LOC_Os02g16830  Glu | -1.83 | -2.37 | 1.37 | 1.15 | -0.32 | -0.54 | 0.83 | 0.61 | 0.29 | 1.37 | 1.15 | 0.83 | -0.22 | -0.54 | -0.32 |
| LOC_Os02g14600  Glu | -0.65 | -0.05 | -1.60 | 0.54 | 0.18 | 0.60 | -1.00 | 1.14 | 1.32 | -1.60 | 0.54 | 0.72 | 2.14 | 2.32 | 0.18 |
| LOC_Os02g14720  *GW2* | -0.14 | -0.28 | -0.44 | 0.34 | -2.64 | -0.13 | -0.58 | 0.20 | -2.44 | -0.44 | 0.34 | -2.30 | 0.78 | -1.86 | -2.64 |
| LOC_Os04g33740  *GIF1* | -1.22 | 0.45 | -0.21 | 0.07 | -1.22 | 1.67 | 1.46 | 1.74 | 0.52 | -0.21 | 0.07 | -1.15 | 0.28 | -0.94 | -1.22 |
| LOC_Os07g39480  *WRKY78* | 0.10 | -0.95 | 0.01 | 1.19 | -0.97 | -1.04 | -1.03 | 0.15 | -0.82 | 0.01 | 1.19 | 0.22 | 1.18 | 0.21 | -0.97 |
| LOC_Os07g08420  RISBZ1 | -0.94 | 0.00 | -0.21 | -0.10 | 0.30 | 0.94 | 0.72 | 0.84 | 1.14 | -0.21 | -0.10 | 0.20 | 0.12 | 0.41 | 0.30 |
|  | **10 DAF** | |  |  |  |  |  |  |  |  |  |  |  |  |  |
| LOC_Os06g04200  *GBSSI* | 0.07 | 1.98 | 1.64 | 1.40 | -0.27 | 1.91 | 1.57 | 1.34 | -0.34 | -0.34 | -0.58 | -2.25 | -0.23 | -1.91 | -1.67 |
| LOC_Os06g30310  *GWD* | -2.42 | -1.65 | -0.26 | -1.55 | -3.97 | 0.77 | 0.51 | 0.87 | -1.55 | -0.26 | 0.10 | -2.32 | 0.36 | -2.06 | -2.42 |
| LOC_Os05g50380  *OsAGPaseL* | 2.03 | 0.48 | 0.98 | 0.07 | 1.27 | 0.99 | 0.77 | -0.34 | 1.24 | 0.27 | -0.32 | 0.97 | -1.24 | 1.32 | 1.67 |
| LOC_Os08g25734  *OsAGPaseS* | 1.74 | -1.54 | 2.10 | 0.60 | 0.95 | -3.28 | -1.18 | -2.68 | -1.73 | 2.10 | 0.60 | 1.55 | -1.50 | -0.55 | 0.95 |
| LOC_Os07g11910  PROLM20 | 0.11 | -1.29 | 1.95 | 0.16 | 0.80 | -1.39 | 0.55 | -1.23 | -0.43 | 1.95 | 0.16 | 0.96 | -1.79 | -0.98 | 0.80 |
| LOC_Os07g10570  PROLM25 | -0.36 | 0.03 | 0.19 | -1.96 | 2.18 | 0.38 | 0.57 | -1.58 | 0.60 | 0.19 | -1.96 | 0.22 | -2.15 | 0.03 | 2.18 |
| LOC_Os12g17010  PROLM29 | -1.30 | -0.31 | -0.76 | -0.30 | 1.06 | 0.99 | 0.23 | 0.69 | 1.75 | -0.76 | -0.30 | 0.76 | 0.46 | 1.51 | 1.06 |
| LOC_Os12g16880  PROLM27 | -0.31 | 0.91 | 1.07 | -0.63 | 0.13 | 1.22 | 2.28 | 0.58 | 0.71 | 1.07 | -0.63 | -0.50 | -1.70 | -1.57 | 0.13 |
| LOC_Os02g16830  Glu | -0.05 | -0.24 | 0.06 | 0.44 | -0.99 | -0.19 | -0.13 | 0.24 | -0.74 | 0.06 | 0.44 | -0.55 | 0.37 | -0.61 | -0.99 |
| LOC_Os02g14600  Glu | -0.71 | 0.18 | 0.76 | -0.33 | 0.02 | 0.89 | 1.65 | 0.56 | 0.58 | 0.76 | -0.33 | -0.32 | -1.09 | -1.08 | 0.02 |
| LOC_Os02g14720  *GW2* | -1.98 | -0.07 | -2.10 | -0.13 | -0.14 | 1.91 | -0.20 | 1.78 | 1.64 | -2.10 | -0.13 | -0.27 | 1.97 | 1.83 | -0.14 |
| LOC_Os04g33740  *GIF1* | -0.73 | 0.79 | -0.33 | 0.22 | -0.72 | 1.52 | 1.19 | 1.75 | 1.03 | -0.33 | 0.22 | -0.49 | 0.56 | -0.16 | -0.72 |
| LOC_Os07g39480  *WRKY78* | 1.94 | 1.01 | 0.77 | 0.43 | 0.19 | -0.93 | -0.15 | -0.49 | -0.31 | 0.77 | 0.43 | 0.62 | -0.34 | -0.15 | 0.19 |
| LOC_Os07g08420  RISBZ1 | -0.89 | 0.02 | -0.26 | 0.17 | 0.30 | 0.91 | 0.65 | 1.08 | 1.38 | -0.26 | 0.17 | 0.47 | 0.43 | 0.73 | 0.30 |
|  | **14 DAF** | |  |  |  |  |  |  |  |  |  |  |  |  |  |
| LOC_Os06g04200  *GBSSI* | 0.97 | 3.68 | 3.41 | 3.04 | 0.64 | 2.71 | 2.44 | 2.07 | -0.33 | -0.27 | -0.64 | -3.04 | -0.38 | -2.77 | -2.40 |
| LOC_Os06g30310  *GWD* | -0.04 | -0.55 | 0.80 | -0.48 | 0.79 | -0.51 | 0.84 | -0.44 | 0.82 | 1.35 | 0.07 | 1.33 | -1.28 | -0.01 | 1.27 |
| LOC_Os05g50380  *OsAGPaseL* | 1.03 | 0.25 | 0.83 | 0.31 | 1.83 | 0.17 | 0.21 | -1.66 | 0.75 | 0.12 | -0.88 | 1.12 | -0.32 | 1.13 | 1.75 |
| LOC_Os08g25734  *OsAGPaseS* | 1.42 | 0.80 | 0.77 | -0.35 | 0.52 | -0.62 | -0.64 | -1.77 | -0.90 | -0.02 | -1.15 | -0.28 | -1.13 | -0.26 | 0.87 |
| LOC_Os07g11910  PROLM20 | 0.53 | -1.58 | 0.15 | -1.34 | 0.36 | -2.11 | -0.39 | -1.88 | -0.18 | 1.72 | 0.23 | 1.93 | -1.49 | 0.21 | 1.70 |
| LOC_Os07g10570  PROLM25 | 0.04 | -0.09 | 0.62 | -0.07 | 2.74 | -0.13 | 0.59 | -0.10 | 2.71 | 0.71 | 0.02 | 2.83 | -0.69 | 2.12 | 2.81 |
| LOC_Os12g17010  PROLM29 | -3.22 | -1.22 | 2.37 | -2.99 | -2.57 | 2.00 | 5.59 | 0.23 | 0.65 | 3.59 | -1.77 | -1.35 | -5.36 | -4.94 | 0.42 |
| LOC_Os12g16880  PROLM27 | -2.54 | -0.32 | 2.08 | -2.35 | 0.06 | 2.22 | 4.62 | 0.19 | 2.60 | 2.40 | -2.03 | 0.38 | -4.43 | -2.02 | 2.41 |
| LOC_Os02g16830  Glu | -1.03 | 0.00 | 1.81 | -0.51 | -0.11 | 1.03 | 2.84 | 0.52 | 0.92 | 1.81 | -0.51 | -0.11 | -2.32 | -1.92 | 0.40 |
| LOC_Os02g14600  Glu | -0.54 | 1.22 | 0.88 | 0.62 | 0.35 | 1.76 | 1.42 | 1.17 | 0.90 | -0.34 | -0.59 | -0.86 | -0.25 | -0.52 | -0.27 |
| LOC_Os02g14720  *GW2* | 0.05 | 0.65 | 0.82 | 0.28 | 0.29 | 0.59 | 0.77 | 0.23 | 0.24 | 0.17 | -0.36 | -0.36 | -0.54 | -0.53 | 0.01 |
| LOC_Os04g33740  *GIF1* | -0.12 | 0.34 | 1.39 | -0.95 | -0.13 | 0.46 | 1.50 | -0.83 | -0.02 | 1.05 | -1.29 | -0.47 | -2.34 | -1.52 | 0.82 |
| LOC_Os07g39480  *WRKY78* | -0.26 | -0.06 | -0.08 | -0.60 | -1.95 | 0.21 | 0.19 | -0.34 | -1.69 | -0.02 | -0.55 | -1.90 | -0.53 | -1.88 | -1.35 |
| LOC_Os07g08420  RISBZ1 | 0.31 | 0.56 | 0.36 | -0.06 | 1.10 | 0.25 | 0.05 | -0.37 | 0.79 | -0.20 | -0.62 | 0.54 | -0.42 | 0.74 | 1.16 |

ARB – Arborio, BAL – Balilla, CAR – Carnaroli, GV – Gigante Vercelli, VN – Vialone Nano, VOL – Volano.
