# Supplementary material for: Deep sequencing transcriptional fingerprinting of rice kernels for dissecting grain quality traits
Source: BMC Genomics. 2015 Dec 21;16:1091. doi: 10.1186/s12864-015-2321-7 (PMC4687084; doi:10.1186/s12864-015-2321-7)
Supplement: Additional file 8: — qRT-PCR results for the housekeeping genes, showing the best standard curves, selected among the genes indicated by Jain et al. (2006) [ 118 ]. (DOCX 14 kb) [file 12864_2015_2321_MOESM8_ESM.docx]

**Additional file 8:** qRT-PCR results for the best housekeeping genes, showing the best standard curves, selected among the genes indicated by Jain e*t al.* (2006) [61]. Three technical replicates were performed for each sample. Cycle threshold (Ct) values represent the mean values obtained for the three technical replicates. Standard deviations (STDV) among the technical replicates and samples, total means, variation coefficients (CV) and stability coefficients obtained by the NormFinder software (http://moma.dk/normfinder-software) are reported.

| **Gene** | ***ACT11*** | | ***UBC*** | | ***GAPDH*** | | ***UBQ5*** | | ***18S rRNA*** | |
| --- | --- | --- | --- | --- | --- | --- | --- | --- | --- | --- |
| **Sample** | **Ct** | **STDV** | **Ct** | **STDV** | **Ct** | **STDV** | **Ct** | **STDV** | **Ct** | **STDV** |
| **ARB rep1** | 20.36 | 0.05 | 19.21 | 0.04 | 21.24 | 0.11 | 20.78 | 0.07 | 11.13 | 0.04 |
| **ARB rep2** | 19.63 | 0.02 | 19.23 | 0.04 | 21.2 | 0.09 | 20.97 | 0.05 | 12 | 0.01 |
| **ARB rep3** | 20.6 | 0.04 | 19.27 | 0.03 | 19.25 | 0.19 | 22.54 | 0.08 | 11.27 | 0.01 |
| **BAL rep1** | 20.16 | 0.01 | 19.28 | 0.03 | 21.07 | 0.19 | 21.39 | 0.09 | 12.21 | 0.04 |
| **BAL rep2** | 20.26 | 0.02 | 19.31 | 0.05 | 20.94 | 0.05 | 21.5 | 0.04 | 11.21 | 0.09 |
| **BAL rep3** | 20.2 | 0.08 | 19.15 | 0.04 | 19.45 | 0.05 | 20.43 | 0.05 | 12.03 | 0.01 |
| **CAR rep1** | 20.42 | 0.03 | 19.25 | 0.05 | 19.98 | 0.03 | 18.94 | 0.02 | 12.94 | 0.07 |
| **CAR rep2** | 19.73 | 0.05 | 18.98 | 0.03 | 20.35 | 0.03 | 19.02 | 0.14 | 12.58 | 0.02 |
| **CAR rep3** | 19.89 | 0.04 | 19.18 | 0.04 | 20.83 | 0.08 | 21.33 | 0.05 | 11.87 | 0.04 |
| **GV rep1** | 20.21 | 0.07 | 19.3 | 0.04 | 19.89 | 0.24 | 21.49 | 0.06 | 12.21 | 0.12 |
| **GV rep2** | 20.07 | 0.02 | 19.25 | 0.05 | 21.27 | 0.02 | 20.35 | 0.07 | 11.26 | 0.12 |
| **GV rep3** | 20.59 | 0.04 | 19.13 | 0.04 | 20.94 | 0.05 | 20.6 | 0.06 | 12.24 | 0.19 |
| **VN rep1** | 20.36 | 0.05 | 19.24 | 0.06 | 20.98 | 0.03 | 19.92 | 0.04 | 11.31 | 0.04 |
| **VN rep2** | 20.15 | 0.02 | 19.02 | 0.03 | 20.86 | 0.16 | 21.18 | 0.08 | 12.23 | 0.08 |
| **VN rep3** | 20.19 | 0.09 | 18.73 | 0.04 | 20.19 | 0.01 | 20.35 | 0.22 | 11.39 | 0.12 |
| **VOL rep1** | 20.27 | 0.06 | 19.25 | 0.02 | 19.75 | 0.04 | 20.34 | 0.16 | 12.04 | 0.11 |
| **VOL rep2** | 20.43 | 0.01 | 19.21 | 0.04 | 21.15 | 0.08 | 21.19 | 0.07 | 11.24 | 0.08 |
| **VOL rep3** | 20.03 | 0.02 | 19.29 | 0.03 | 20.19 | 0.06 | 19.83 | 0.02 | 12.45 | 0.21 |
| **Total mean** | 20.2 | | 19.18 | | 20.53 | | 20.68 | | 11.87 | |
| **Total STDV** | 0.26 | | 0.15 | | 0.66 | | 0.9 | | 0.55 | |
| **CV** | 0.013 | | 0.007 | | 0.032 | | 0.044 | | 0.046 | |
| **NormFinder Stability value** | 0.002 | | 0.002 | | 0.011 | | 0.016 | | 0.022 | |

ARB = Arborio, BAL = Balilla, CAR = Carnaroli, GV = Gigante Vercelli, VN = Vialone Nano, VOL = Volano, rep = biological replicate, Ct = cycle threshold, STDV = standard deviation, CV = variation coefficient.
