# Supplementary material for: Deep sequencing transcriptional fingerprinting of rice kernels for dissecting grain quality traits
Source: BMC Genomics. 2015 Dec 21;16:1091. doi: 10.1186/s12864-015-2321-7 (PMC4687084; doi:10.1186/s12864-015-2321-7)
Supplement: Additional file 9: — Sequence of the primer pairs used for each locus analysed by qRT-PCRs. (DOCX 12 kb) [file 12864_2015_2321_MOESM9_ESM.docx]

**Additional file 9:** Sequence of the primer pairs used for each *locus* analysed by qRT-PCRs.

| ***Locus*** | **Primer pair** | **Sequence** |
| --- | --- | --- |
| LOC_Os06g04200 | LOC_Os06g04200fw | ACCAGTACAAGGACGCTTGG |
|  | LOC_Os06g04200rev | GCTCCTACCTCAGCCACAAC |
| LOC_Os05g50380 | LOC_Os05g50380fw | GAGGGTAAAGCATGCGTGAG |
|  | LOC_Os05g50380rev | ACAGCAGCAACCTCATTTGG |
| LOC_Os08g25734 | LOC_Os08g25734fw | GACCGGTCTGCTCCAATTTA |
|  | LOC_Os08g25734rev | ATGCCACCTTTTTCACCAAG |
| LOC_Os06g30310 | LOC_Os06g30310fw | CACTGGGCGTTGTCAAAAGA |
|  | LOC_Os06g30310rev | TCAGTACAGGCCCACATTGT |
| LOC_Os07g11910 | LOC_Os07g11910fw | TCCTGCTACAGCAACAGGTG |
|  | LOC_Os07g11910rev | AGGTGTAGCTGGTGCGCTAT |
| LOC_Os04g10530 | LOC_Os04g10530fw | CAACGGGAGAGTCTTCTTCG |
|  | LOC_Os04g10530rev | GGCATCATTCGAACTTTGGT |
| LOC_Os12g17010 | LOC_Os12g17010fw | GCCCTAAACTTGCCGTCAAT |
|  | LOC_Os12g17010rev | ACGGTGGGAATGCTATAGGG |
| LOC_Os12g16880 | LOC_Os12g16880fw | CCATGCGGTGAGTTCGTAAG |
|  | LOC_Os12g16880rev | GGGTGATTGGAAGAAGGGGA |
| LOC_Os02g16830 | LOC_Os02g16830fw | TCAATCGAGCAACACTCTGG |
|  | LOC_Os02g16830rev | TCCTGCTGTTGTGCTTGTTC |
| LOC_Os02g14600 | LOC_Os02g14600fw | GGCAAGGAGATGTTGTTGCA |
|  | LOC_Os02g14600rev | TGAGGGGTTGGACACTTGTT |
| LOC_Os02g14720 | LOC_Os02g14720fw | TTGCAGCACATCCTTTTCAG |
|  | LOC_Os02g14720rev | GCCACAGACAGGATTCCCTA |
| LOC_Os04g33740 | LOC_Os04g33740fw | CTCTGAGGAGCCTGATCGAC |
|  | LOC_Os04g33740rev | AGGCTCCATTCATCATGACC |
| LOC_Os07g39480 | LOC_Os07g39480fw | AAGCCTGAATACTGCCCTGT |
|  | LOC_Os07g39480rev | GCCCATATTTGCGCCAGTTA |
| LOC_Os07g08420 | LOC_Os07g08420fw | AGGGGCTAGACAACTTCACC |
|  | LOC_Os07g08420rev | CGCCACCACAAGCTCTATTC |
| *UBC* | *UBC*fw | CCGTTTGTAGAGCCATAATTGCA |
|  | *UBC*rev | AGGTTGCCTGAGTCACAGTTAAGTG |
